# Crystal Facet‐Controlled Efficient SnS Photocathodes for High Performance Bias‐Free Solar Water Splitting

**DOI:** 10.1002/advs.202102458

**Published:** 2021-09-08

**Authors:** Hyungsoo Lee, Jin Wook Yang, Jeiwan Tan, Jaemin Park, Sang Gi Shim, Young Sun Park, Juwon Yun, Kyungmin Kim, Ho Won Jang, Jooho Moon

**Affiliations:** ^1^ Department of Materials Science and Engineering Yonsei University Seoul 03722 Republic of Korea; ^2^ Department of Materials Science and Engineering, Research Institute of Advanced Materials Seoul National University Seoul 08826 Republic of Korea

**Keywords:** facet control, fast crystallization, molecular ink, photoelectrochemical water splitting, SnS, tandem device

## Abstract

To achieve a high solar‐to‐hydrogen (STH) conversion efficiency, delicate strategies toward high photocurrent together with sufficient onset potential should be developed. Herein, an SnS semiconductor is reported as a high‐performance photocathode. Use of proper sulfur precursor having weak dipole moment allows to obtain high‐quality dense SnS nanoplates with enlarged favorable crystallographic facet, while suppressing inevitable anisotropic growth. Furthermore, the introducing Ga_2_O_3_ layer between SnS and TiO_2_ in SnS photocathodes efficiently improves the charge transport kinetics without charge trapping. The SnS photocathode reveals the highest photocurrent density of 28 mA cm^−2^ at 0 V versus the reversible hydrogen electrode. Overall solar water splitting is demonstrated for the first time by combining the optimized SnS photocathode with a Mo:BiVO_4_ photoanode, achieving a STH efficiency of 1.7% and long‐term stability of 24 h. High performance and low‐cost SnS photocathode represent a promising new material in the field of photoelectrochemical solar water splitting.

## Graphical abstract

Crystal facet control dramatically enhances both the photocurrent density of SnS photocathodes to 28 mA cm^−2^ at 0 V_RHE_ and the onset potential to 0.4 V_RHE_ owing to the extended (101) facet. By combining the photocathode with a Mo:BiVO_4_ photoanode, unbiased solar water splitting is successfully demonstrated with bench‐mark efficiency (≈1.7%) and long‐term stability of 24 h.

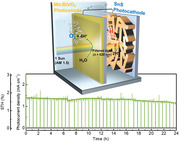

## 1 Introduction

Simultaneous conversion and storage of abundant but intermittent solarenergy have been proposed as a sustainable approach for producing clean and renewable fuels. Photoelectrochemical (PEC) water splitting can provide an attractive and efficient platform to generate ecofriendly hydrogen fuel.^[^ ^1^ ^]^ The solar‐to‐hydrogen (STH) efficiency of PEC devices using photovoltaic grade III–V semiconductors has nearly approached the theoretical maximum.^[^ ^2^, ^3^ ^]^ However, these costly PEC devices need to be fabricated through complicated and time‐consuming processes.^[^ ^4^ ^]^ To produce sustainable hydrogen for a scale commensurate with the global energy demand, low‐cost and easily processable semiconductors with desirable optoelectronic properties for PEC water splitting should be explored.^[^ ^5^, ^6^, ^7^ ^]^ According to the theoretical calculation on a D4‐type tandem device (dual light absorber: four photons to one hydrogen), a band gap (*E*_g_) of 1.1–1.3 eV for the bottom electrode is required for realizing STH efficiencies of ≈24% when the top electrode has *E*_g_ of 1.7–1.8 eV.^[^ ^8^, ^9^ ^]^ In this regard, most cost‐competitive light absorbers for water splitting, such as TiO_2_ (*E*_g_ ≈ 3.2 eV),^[^ ^10^ ^]^ BiVO_4_ (*E*_g_ ≈ 2.4 eV),^[^ ^11^ ^]^ Cu_2_BaSnS_4_ (*E*_g_ ≈ 2.0 eV)^[^ ^12^ ^]^ and Cu_2_O (*E*_g_ ≈ 2.0 eV),^[^ ^13^ ^]^ are considered unsuitable for bottom photoelectrodes due to their large band gap. Therefore, several low band gap semiconductors for PEC photocathodes, such as CuFeO_2_,^[^ ^14^ ^]^ CuBi_2_O_4_,^[^ ^15^ ^]^ CuSbSe_2_,^[^ ^16^ ^]^ Cu_2_ZnSn(S,Se)_4_,^[^ ^17^ ^]^ and CuSbS_2_,^[^ ^18^ ^]^ have been researched extensively. However, most of these alternative absorber materials suffer from instability against photocorrosion, low carrier mobility, and unavoidable secondary phase formation due to a narrow stoichiometric window.^[^ ^19^ ^]^

SnS has recently emerged as an unexplored promising semiconductor for PEC water splitting owing to its beneficial optoelectrical properties such as appropriate carrier concentrations (10^14^ − 10^17^ cm^−3^), high light absorption coefficient (>10^5^ cm^−1^) in visible region, and small *E*_g_ (1.2–1.3 eV).^[^ ^20^ ^]^ In addition, pure‐phase SnS is readily obtainable through facile constituent ratio control in a precursor solution without forming any detrimental impurity phase, unlike the multinary Cu chalcogenide and kesterite.^[^ ^21^ ^]^ Similar to Sb_2_Se_3_, the control of the SnS crystal orientation plays a pivotal role in efficient light harvesting. It has been reported that [101]‐oriented SnS exhibits excellent carrier mobility (≈90 cm^2^ V^−1^ s^−1^).^[^ ^22^ ^]^ From the photocathode based on well‐oriented SnS nanoplate, our recent work demonstrated high photocurrent density of −19 mA cm^−2^ at 0 V versus the reversible hydrogen electrode (RHE).^[^ ^21^ ^]^ However, it is still far below the theoretical maximum (≈40 mA cm^−2^ assuming 100% incident photon‐to‐current efficiency (IPCE) for an absorber with *E*_g_ of 1.2 eV).^[^ ^23^ ^]^ Moreover, the onset potential and fill factor of the SnS photocathode device need to be further improved to realize unassisted overall PEC water splitting.

SnS has an anisotropic 2D orthorhombic crystal structure with in‐plane covalent bonds along the [101] direction and van der Waals bonds along the [010] direction.^[^ ^24^, ^25^ ^]^ The charge carriers hop across the van der Waals‐bonded in a [010] direction of 2D SnS with a relatively high resistance, whereas the charge carriers were readily collected in a [101] direction through the covalent bonds of 2D SnS owing to higher conductivity. Consequently, the carrier transport in SnS along the [101] direction is more efficient than that along the [010] direction due to its anisotropic crystallographic nature.^[^ ^24^ ^]^ In such a 2D semiconductor‐based photocathode, when the (010) plane in SnS is junctioned with an n‐type overlayer, recombination of photogenerated charges likely occurs due to facile hopping over the van der Waals bonds, resulting in a significant decrease in the fill factor.^[^ ^26^ ^]^ The reduced fill factor is generally accompanied by loss in the photocurrent and onset potential. In this regard, 2D SnS should be oriented along the (101) planes where in‐plane covalent bonds are present, with excellent charge transport kinetics when forming the *p*‐*n* junction to ensure separation of the photogenerated charges.^[^ ^27^ ^]^ However, the crystallographic habit of 2D SnS likely gives rise to thin platelets with the (010) basal plane during the crystal growth, which makes it difficult for them to be vertically aligned with the preferred (101) facet‐oriented.^[^ ^27^ ^]^ Therefore, it is necessary to develop a methodology to synthesize thick nanoplates with the extended (101) facets from which the SnS crystals can be vertically oriented while exposing the desirable (101) planes.

Herein, we propose kinetically favored approach to control the crystal facets of the SnS nanoplates by employing different precursors with varying polarizations. The crystal growth of SnS is controlled by the dipole moment difference of a sulfur precursor, resulting in well‐oriented SnS nanoplates with enlarged (101) facets. The resulting crystal facet‐controlled SnS‐based photocathodes revealed a remarkable onset potential of 0.3 V_RHE_ and photocurrent density of 23 A cm^−2^ at 0 V_RHE_. In addition, interface engineering by introduction of an *n*‐type Ga_2_O_3_ overlayer with a high energy conduction band minimum (CBM) enabled bending of the energy level of SnS at the interface, further boosting the onset potential to 0.4 V_RHE_. We, for the first time, demonstrated a D4 tandem device consisting of a photoanode (Mo:BiVO_4_) and a photocathode (SnS) from which unassisted overall water splitting was accomplished in a borate buffer solution, revealing an STH efficiency of 1.7% as well as stable operation for 24 h. Our SnS‐based PEC tandem device opened an unexplored path for realizing practical solar hydrogen production in an efficient and cost‐competitive manner.

## 2 Results and Discussion

### 2.1 Synthesis of SnS Nanoplates with Extended Favorable Facets

**Figure 1:**
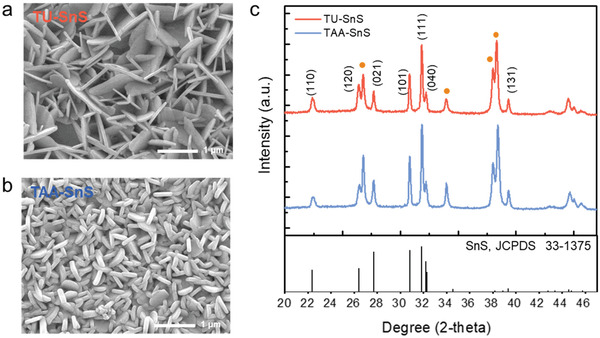
Top‐view SEM images of the SnS obtained using the inks with different precursors of a) TU and b) TAA. c) XRD patterns of three different SnS layers with varying Sn/S ratios of 1.0, 1.2, and 1.5. The reference diffraction patterns of SnS (JCPDS no. 33‐1375) are included for comparison purpose. The orange peaks represent the diffraction pattern of Au/FTO.

The SnS nanoplate with the (101) facet tends to grow sparsely due to its anisotropic crystal nature.^[^ ^27^ ^]^ To extend the (101) facet with a high surface energy, the growth of the thermodynamically favorable plane should be suppressed. We tried to kinetically restrain the favorable growth of the SnS (010) plane by employing sulfur precursors with different molecular polarities. Thiourea (TU), which is a typical precursor used to synthesize the multicomponent chalcogenide compounds, thioacetamide (TAA), and L‐cysteine were selected for this study.^[^ ^28^ ^]^ The SnS molecular inks were prepared by dissolving the sulfur precursor and SnCl_2_ to 2‐methoxyethanol (2ME) in a Sn/S molar ratio of 1.5, which enabled the fabrication of defect‐free phase‐pure SnS.^[^ ^21^ ^]^ Molecular inks based on both TU and TAA retained the homogenous solution of transparent yellow color (Figure S1, Supporting Information), whereas adding L‐cysteine to the SnCl_2_‐dissolved solution immediately induced precipitation, forming a dark colloidal ink. It is considered that L‐cysteine having low dipole moments results in a rapid complexation with Sn^2+^ ions, leading to the precipitation of the SnS precursor.^[^ ^29^ ^]^ Thus, SnS absorber layers were fabricated using molecular solutions based on the TU and TAA precursors (denoted as TU‐ink and TAA‐ink, respectively). The morphologies of the resulting two different SnS absorbers were examined using scanning electron microscopy (SEM). Both the samples consisted of 2D, nanoplate‐like particulate layers (**Figure** **1**a,b). The aspect ratio of the TU‐ink‐derived SnS (denoted as TU‐SnS) was higher than that of the TAA‐ink‐derived SnS (denoted as TAA‐SnS), indicating that TAA‐SnS had a thicker plate thickness. The plate thickness and height of the TU‐SnS individual particles were determined using the ImageJ software as 70 ± 10 nm and 1 ± 0.2 µm, respectively. However, TAA‐SnS with a thickness of 90 ± 10 nm and height of 0.3 ± 0.1 µm exhibited a highly dense arrangement of SnS. The TU‐SnS layer had a higher cross‐sectional thickness (600 ± 50 nm, Figure S2a, Supporting Information) than that of compactly grown TAA‐SnS (400 ± 50 nm, Figure S2b, Supporting Information).

Phase identification of the resulting SnS layers was performed via X‐ray diffraction (XRD) measurements, as shown in Figure 1c. Two samples, fabricated with different sulfur precursors of TU and TAA, exhibited similar diffraction patterns, while the peaks of the (101) and (111) facets were dominant in both the SnS layers, revealing that both TU‐SnS and TAA‐SnS were vertically aligned along the [101] direction. The XRD patterns well matched with the powder diffraction measurements for orthorhombic SnS (JCPDS No. 33‐1375). No diffraction peaks indicating secondary phases, such as Sn_2_S_3_ and SnS_2_, were observed.^[^ ^24^ ^]^ Raman spectroscopy, which is a chemical analysis technique capable of accurately determining vibrational modes, was performed to ensure phase purity of SnS. As shown in Figure S3, Supporting Information, characteristic peaks observed at 95, 161, 183, and 220 cm^−1^ were assigned to SnS. The vibration peaks related to Sn_2_S_3_ and SnS_2_ located at 304 and 313 cm^−1^, respectively, were not observed. Further, Raman analysis confirmed that a phase‐pure SnS absorber can be obtained using TU and TAA as sulfur precursors. TAA‐SnS revealed higher intensities for the (101) and (111) peaks than TU‐SnS. To quantify the preferred orientation, we calculated the texture coefficient *T*_c_ as follows:

(1) $$T_{c}\left(\mathrm{hkl}\right)\;=\;n\frac{I(\mathrm{hkl})/I_{o}\left(\mathrm{hkl}\right)}{\sum_{1}^{n}I(\mathrm{hkl})/I_{o}\left(\mathrm{hkl}\right)},$$

where *I*(*hkl*) represents the measured intensity of the peak corresponding to the *hkl* diffraction, *I*_o_(*hkl*) denotes the intensity for a standard orthorhombic SnS powder sample (JCPDS No. 33‐1375), and *n* is the total number of diffraction peaks used in the calculation. A large *T*_c_ value for a specific diffraction peak indicates the preferred orientation. As shown in Figure S4, Supporting Information, the *T*_c_ values for the (101) and (111) planes of both TU‐SnS and TAA‐SnS were predominant as compared with (110), (120), (021), (131), and (040). in particular, TAA‐SnS exhibited higher *T*_c_ values for the (101) and (111) planes and a lower *T*_c_ value for the (040) plane than TU‐SnS. The texture coefficient analysis indicated that the TAA‐ink enables the development of a densely packed TAA‐SnS nanoplate layer arranged in the preferred orientation, exposing enlarged (101) facets.

**Figure 2:**
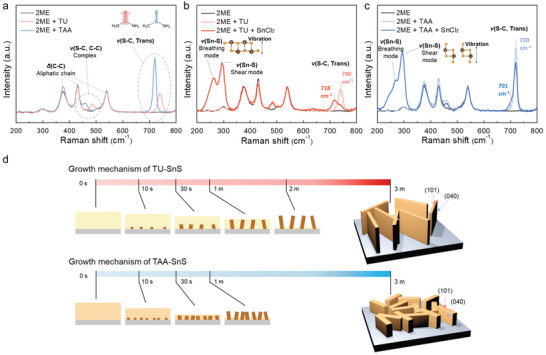
Molecular structures of the TU‐ink and TAA‐ink determined by liquid Raman analysis. Liquid Raman spectra for a) different sulfur precursors containing solution when dissolved in 2ME: b) TU‐ink and c) TAA‐ink. d) Schematic showing the formation process of SnS light‐absorbing layer from the precursor inks with either TU or TAA as a function of annealing time.

To elucidate the origin of different morphology evolutions, it is necessary to understand the molecular chemistry of the TU‐ and TAA‐inks according to the sequential step of ink preparation. The liquid Raman spectra of 2ME and two 2ME−S precursor mixtures (i.e., TU·2ME and TAA·2ME) are shown in **Figure** **2**a. The peaks located at ≈375, ≈426, ≈538 cm^−1^, ascribed to the C−C aliphatic chain stretching vibration in 2ME,^[^ ^30^ ^]^ appeared even after the S precursor was added, whereas the stretching vibrations of S−C appeared at 483 and 740 cm^−1^ for the TU·2ME solution corresponding to S−C/C−C complex stretching and S−C trans conformer, respectively.^[^ ^19^ ^]^ In contrast, the stretching vibrations of S−C for the TAA·2ME solution were red‐shifted at 458 and 720 cm^−1^ with respect to the TU·2ME solution. Upon addition of SnCl_2_ to prepare the TU‐ink, Sn^2+^ reacted with sulfur in TU. The peak corresponding to the stretching mode of the S−C symmetric stretching vibration shifted to 718 cm^−1^ (Figure 2b). In contrast, after the addition of SnCl_2_ into the TAA·2ME solution, as shown in Figure 2c, the S−C symmetric stretching vibration exhibited lower Raman shift at 701 cm^−1^. Significant red‐shifting of the S−C stretching frequency in the TAA‐ink indicates a significant decrease in the force constant owing to a low bond strength between sulfur and carbon as compared to that in the TU‐ink (inset of Figure 2a). This indicates that TAA has weaker dipole moments than TU.^[^ ^31^ ^]^ In both TU‐SnS and TAA‐SnS, the peaks corresponding to the breathing and shear vibrational mode of Sn−S appeared at ≈260 and ≈300 cm^−1^, respectively.^[^ ^32^, ^33^ ^]^ The breathing vibrational mode representing the vibrations along the [010] direction was clearly observed in the TU‐ink (inset of Figure 2b), whereas it was hardly detected in the TAA‐ink.^[^ ^21^ ^]^ Instead, the TAA‐ink revealed a high intensity of shear vibrational mode corresponding to the vibrations along the [101] direction (inset of Figure 2c). Owing to the strong dipole moment of S in the TU‐ink, the Sn^2+^ ions aggregated into Sn−S long chains in the molecular ink, as indicated by the breathing vibrational mode. By contrast, there is likely to be less aggregation of the Sn−S chains due to the weak dipole moment of S in the TAA‐ink; therefore, the breathing vibrational mode is not observed, while the shear mode is clearly detected. It is hypothesized that the aggregated Sn−S long chains due to the strong dipole moment‐driven van der Waals bonds exhibit the (040) habit orientation in the TU‐ink, whereas the weak dipole moment induced Sn−S short chains retain the (101) habit in the TAA‐ink.

The plausible SnS formation mechanisms of the two different SnS structures depending on the ink types are schematically depicted in Figure 2d. To explain the growth mechanism of two different SnS structures, the thermodynamic principle suggests that the differences in the nuclei number as well as the grain size between the TU‐ and TAA‐ink‐derived SnS depend on the nucleation rate. The nucleation rate and growth can be quantitatively expressed by an Arrhenius‐type equation according to the classical nucleation theory, which describes the negative temperature dependence of the nucleation rate as follows.^[^ ^34^, ^35^ ^]^

(2) $$\frac{\mathrm{dN}}{\mathrm{dt}}\;=\;A\;\exp\left(-\frac{\Delta G^{*}}{k_{B}T}\right)\;=\;A\;\exp\left(-\frac{16\pi \gamma^{3}V_{M}^{2}}{{3k}_{B}^{3}T^{3}N_{A}^{2}{\left(\mathrm{lnS}\right)}^{2}}\right),$$

where *N*, *A*, *k*_B_, T are the number of nuclei, pre‐exponential factor, Boltzmann constant, and temperature, respectively. The critical Gibbs free energy of nucleation (ΔG*) can be represented as a function of the surface energy *γ*, molar volume of particle *V*_M_, and level of supersaturation S. Since two molecular inks are based on the 2ME solvent with the same concentration and identical annealing temperature, *V*_M_ for both the TAA‐ink and TU‐ink will be similar owing to the indistinguishable molecular size difference between TAA and TU. However, TAA has a relatively lower solubility in 2ME than TU due to its low polarity. The molecular ink based on TAA likely increases the degree of supersaturation, which in turn decreases the critical Gibbs free energy of nucleation. The reduced critical Gibbs free energy accelerates the nucleation rate, exponentially increasing the number of nuclei formed per unit volume during the initial growth stage. As shown at the bottom of Figure 2d, the precursor film obtained in the TAA‐ink showed plenty of SnS nuclei upon annealing as the TAA‐ink immediately evaporated within 1 min upon annealing. Each nucleus grows homogeneously until the precursor ink turns into the SnS nanoplates. According to the liquid Raman spectra, most of the Sn−S molecules were found to be already oriented as the (101) habit in the molecular ink state. After 3 min‐annealing, rapid nucleation and growth resulted in a large number of TAA‐SnS nanoplates with the preferred oriented (101) facets. However, the increased Gibbs free energy reduced the number of nuclei and decelerated the nucleation rate. TU‐SnS slowly evaporated when annealed for double the annealing time and low SnS nuclei growth was observed, as shown at the top in Figure 2d. The Sn−S molecules in the TU‐ink exist as the (040) habit in the molecular ink state. A slow nucleation event results in the particles to grow into thermodynamically stable facets such as (040). Thus, the thermodynamically unstable (101) facet disappears, resulting in narrow (101) facet‐oriented in TU‐SnS nanoplates. In contrast, the fast nucleation of numerous nuclei leads to a kinetically regulated and dense growth of the TAA‐SnS nanoplates with an enlarged (101) facet.

### 2.2 PEC Performance of Facet‐Controlled SnS Photocathodes via Molecular Ink

**Figure 3:**
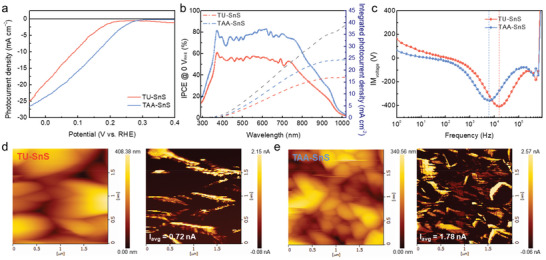
a) *J*–*V* curves and b) IPCE spectra of Pt/TiO_2_/CdS/SnS/Au/FTO photocathodes using TU‐SnS (red) and TAA‐SnS (blue) under solar simulated AM 1.5G irradiation in 0.5 m H_2_SO_4_ electrolyte (pH ≈ 1). Theoretical maximum photocurrent density (*J*_max_) assuming 100% IPCE is represented by the black dotted line. c) Bode plot of the IMVS for TU‐ and TAA‐SnS based photocathodes. Topography and current mapping of d) TU‐SnS and e) TAA‐SnS without overlayers were revealed via c‐AFM.

**Figure 4:**
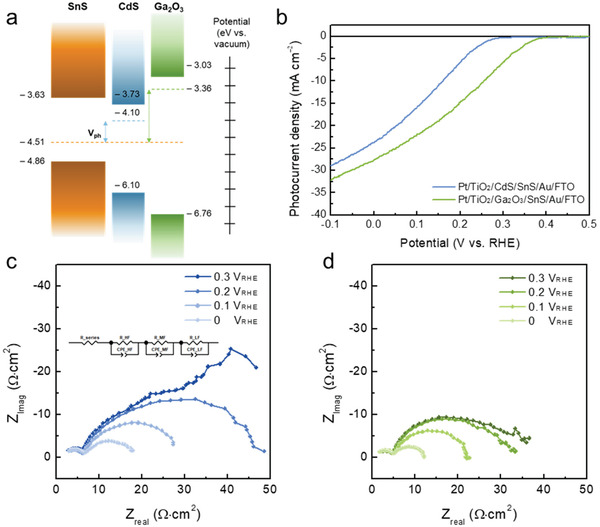
a) Energetics schematic showing the band energy positions for the SnS with either CdS or Ga_2_O_3_ overlayer calculated from UPS data. b) *J*–*V* curves of Pt/TiO_2_/CdS/SnS/Au/FTO (blue) and Pt/TiO_2_/Ga_2_O_3_/SnS/Au/FTO (green) photocathodes under solar simulated AM 1.5G irradiation in 0.5 m H_2_SO_4_ electrolyte (pH ≈ 1). c) Nyquist plots of the EIS spectra for two different types of SnS photocathodes under the same electrolyte conditions.

**Figure** **3**a shows the PEC performance of Pt/TiO_2_/CdS/SnS/Au/fluorine‐doped tin oxide (FTO) photocathodes using either the TU‐ or TAA‐ink‐derived SnS absorber in a 0.5 m H_2_SO_4_ (pH ≈ 1) electrolyte. The Pt catalytic layer was deposited by the sputtering method, while atomic layer deposition (ALD) was used for the TiO_2_ layer. The CdS layer was deposited by the chemical bath deposition method to obtain a thin conformal layer, according to a previous study.^[^ ^21^ ^]^ The onset potential of the TAA‐SnS‐based photocathodes (≈0.3 V_RHE_) shifted toward a positive direction as compared to the TU‐SnS‐based photocathodes (≈0.23 V_RHE_). The photocurrent density of the TAA‐SnS device approached 23.5 mA cm^−2^ at 0 V_RHE_, which is the highest value obtained so far for the SnS photocathode. Note that the data shown in Figure 3a represent the best‐performing device, whereas a photocurrent density of 20–23 mA cm^−2^ at 0 V_RHE_ was observed. While the TAA‐SnS photocathode revealed a remarkable photocurrent density, the photocurrent density of the TU‐SnS device was relatively low (≈19 mA cm^−2^ at 0 V_RHE_). Figure S5, Supporting Information shows the applied bias photon‐to‐current efficiency (ABPE) calculated from Figure 3a according to the equation ABPE = *I*_ph_ × (*E*_RHE_ − *E*_H+/H2_)/*P*_SUN_ × 100%, where *I*_ph_ is the photocurrent density obtained under an applied bias of *E*_RHE_, *E*_H+/H2_ is 0 V_RHE_, and *P*_SUN_ is 100 mW cm^−2^. It is noticeable that the maximum value of the ABPE for the TAA‐SnS device is 1.70% at a more positive potential of 0.115 V_RHE_ as compared with the TU‐SnS device (0.09 V_RHE_). *J*–*V* curve and ABPE of TU‐SnS device show secondary peaks in the range of 0.3−0.4 V_RHE_. It was confirmed that this pre‐reduction current peak contributes to the non‐faradaic reaction occurred at the interface of CdS/TU‐SnS (e.g., the reduction of interface states), hampering charge separation. On the other hand, as shown in Figure 3a (blue line) and **Figure** **4**b (green line), the disappearance of the pre‐reduction peak after introduction of the TAA‐SnS absorber layer instead of TU‐SnS is attributed to the reduced defect sites due to the extension of desirable (101) facets. It is indicated that the fast nucleation strategy is advantageous not only for increasing the fill factor, but also for enhancing both the photocurrent and onset potential. As shown in Figure 3b, both the TU‐ and TAA‐SnS‐based photocathodes were able to harvest photons near 1000 nm; however, the IPCE was much higher for the TAA‐SnS device in the entire wavelength. The photon flux that TU‐ and TAA‐SnS is possible to absorb is shown in Figure S6, Supporting Information. By integrating the photon flux absorbed by SnS, TU‐ and TAA‐SnS devices show the photocurrent density of 17.5 and 24 mA cm^−2^, respectively, which coincides with the photocurrent of the *J*–*V* curve (Figure 3b). The optical properties (e.g., absorbance and band gap of SnS/FTO samples) for TU‐ and TAA‐SnS were nearly identical (Figure S7, Supporting Information), hence the maximum photocurrent densities of both TU‐ and TAA‐SnS devices could reach ≈32 mA cm^−2^, assuming 100% absorbed photon‐to‐current conversion efficiency (APCE) (Figure S8, Supporting Information). The high IPCE and APCE values for the TAA‐SnS device do not originate from better light absorption. Therefore, the enhanced light absorption can be excluded from the potential origin of the performance difference.

To understand the performance difference observed for the TU‐ and TAA‐SnS devices, the band position of the SnS samples was investigated by ultraviolet photoelectron spectroscopy (UPS), as shown in Figure S9a, Supporting Information. The Fermi level of TAA‐SnS was calculated by UPS as −4.51 eV versus vacuum (detailed calculation methods are described in Experimental Section), which is lower than that of TU‐SnS (−4.26 eV versus vacuum). Therefore, the large difference between the Fermi level of TAA‐SnS and n‐type CdS induces a much higher photovoltage when the buried junction is formed with an *n*‐type overlayer. The surface electronic structures of low‐dimensional semiconductors depend on the crystal facet; it is speculated that dangling bonds the along [101] orientation are expected to have a high surface energy and high photocatalytic activity.^[^ ^36^, ^37^ ^]^ The improvement of the photovoltage can be verified by obtaining flat band potential (*V*_fb_). Mott−Schottky plot is useful for determining the *V*_fb_ and the acceptor concentration (*N*_A_) by measuring the capacitance formed at the semiconductor−electrolyte contact as a function of the applied potential. As shown in Figure S10, Supporting Information, we determined Mott–Schottky plot. While the calculated *N*_A_ (9.56 × 10^16^ cm^−3^) of the TAA‐SnS is similar to that of TU‐SnS (9.37 × 10^16^ cm^−3^), the *V*_fb_ of TAA‐SnS is 0.28 V, which is 0.07 V higher than that of TU‐SnS. This indicates that higher photovoltage is able to develop when TAA‐SnS is used as an absorber layer.

To further elucidate the surface charge kinetics in the SnS photocathodes, we performed intensity‐modulated photovoltage spectroscopy (IMVS) to examine the photovoltage variation as a function of light intensity perturbation frequency under the open‐circuit condition. In the Bode plot of IMVS, the minimum value of the imaginary photovoltage can be characterized by the peak in the high‐frequency region (approximately kH range), representing the characteristic rate constant of surface charge recombination (*k*_rec_) in proportion to the characteristic frequency (*f*_rec_). The characteristic time constant (*τ*_rec_) at which recombination occurs was calculated using the equation *τ*_rec_ = (1/*k*_rec_) = (1/2*πf*_rec_). The peak of the TAA‐SnS photocathode shifted toward a low‐frequency range as compared with that in the TU‐SnS photocathode, indicating an increase in *τ*_rec_ from 9.95 to 25.02 µs (Figure 3c). This implies that the surface charge recombination was significantly suppressed in the TAA‐SnS‐based photocathode.

We performed conductive atomic force microscopy (c‐AFM) analysis, which is well known as an efficiency technique to investigate charge carrier transport in nanostructured photoelectrodes. Figure 3d,e show the surface topography and current mapping of the TU‐ and TAA‐SnS nanostructures, respectively. The TU‐SnS nanostructures revealed larger 2D structures and small distribution of current, as shown in Figure 3d. In contrast, the SnS nanostructures derived from the TAA‐ink, as shown on the left of Figure 3e, showed smaller 2D nanoplates in accordance with the SEM images in Figure 1a,b. The TAA‐SnS exhibited a higher average current (*I*_avg_) of 1.78 nA than TU‐SnS (*I*_avg_ = 0.72 nA). Instead, the TU‐SnS sample showed a relatively large region of dark current because of the dominant (040) facet, as shown in the current mapping (right of Figure 3d). This observation indicates that the (040) facets can act as the recombination sites of the photoexcited electrons owing to facile charge hopping across the van der Waals bonds in SnS. In contrast, in the TAA‐SnS sample, the electrical carriers can be readily collected in vertically aligned SnS nanoplates, as revealed by the entirely bright‐yellow‐colored region. Therefore, in conjunction with the IMVS results, we can conclude that the extended (101) facets not only prevent the charge recombination but also enhance the charge carrier separation.

### 2.3 Interface Engineering for Unassisted PEC Water Splitting

Despite the high photocurrent density, the TAA‐SnS photocathode still exhibited a low onset potential to achieve unassisted solar water splitting based on the photocathode–photoanode tandem device. According to the band alignment, as shown in Figure 4a, at the CdS/SnS heterojunction, a small photovoltage (*V*_ph_) was obtained from the quasi‐Fermi level difference at the interface between SnS and CdS. Accordingly, the Pt/TiO_2_/CdS/SnS/Au/FTO photocathode resulted in a low onset potential of 0.3 V_RHE_, which was affected by both *V*_ph_ and overpotential (Figure 4b). In this regard, interface engineering for band tuning is required to improve the photovoltage. For proper band alignment to improve the photovoltage, *n*‐type Ga_2_O_3_ was inserted instead of CdS to facilitate the charge transport. By analyzing the band position of Ga_2_O_3_ via UPS (Figure S11, Supporting Information), we confirmed that a twofold increase in *V*_ph_ can develop between the Ga_2_O_3_/SnS heterojunction (Figure 4a). Upon illuminated equilibrium state, band bending occurs at the SnS/CdS interface, forming an Ohmic contact (Figure S12a, Supporting Information). On the other hand, the Schottky contact forms at the SnS/Ga_2_O_3_ interface (Figure S12b, Supporting Information). Because the CBM of bulk Ga_2_O_3_ is lower than that of bulk SnS, the electrons are able to tunnel and transport to the semiconductor‐liquid junction when very thin Ga_2_O_3_ overlayer is used. In this regard, the thickness of Ga_2_O_3_ layer is a critical factor in determining PEC performance of SnS photocathodes. As shown in Figure S13, Supporting Information, inserting quite thin Ga_2_O_3_ layer of 5 nm is insufficient to enhance the onset potential. When relatively thick Ga_2_O_3_ layer of 20 nm is inserted, it would be difficult for the photogenerated electrons to cross the Schottky barrier. While the onset potential of 20 nm‐thick Ga_2_O_3_ junctioned SnS photocathode is higher than that of the CdS‐modified SnS photocathode, the photocurrent density of Ga_2_O_3_/SnS photocathode tends to be decreased. Therefore, adopting appropriate thickness of Ga_2_O_3_ overlayer (10 nm) enables us to obtain dramatically improved PEC performance in terms of photocurrent and onset potential. It was confirmed that SnS nanoplates were vertically erected to Au/FTO substrate as shown in Figure S14a, Supporting Information. In addition, it was confirmed that 10 and 15 nm of Ga_2_O_3_ and TiO_2_ were uniformly deposited through the ALD method on SnS nanoplates (Figure S14b, Supporting Information). In order to verify that (101) facet‐oriented SnS is vertically grown, high‐resolution TEM (HRTEM) image of SnS layer adjacent to Au/FTO substrate was obtained (Figure S14c, Supporting Information). Figure S14d, Supporting Information shows the magnified image for the selected region (red box in Figure S14c, Supporting Information) to investigate the orientation of the SnS layer, revealing the crystallization of SnS by fast Fourier transform image (Figure S14e, Supporting Information). The horizontally aligned lattices have a spacing of 2.63 Å, corresponding to the out‐of‐plane lattice spacing for the (040) plane along the [010] direction (orange arrow). By contrast, the vertically aligned lattice fringes have a spacing of 1.97 Å, corresponding to the in‐plane lattice spacing for the (002) plane along the [001] direction (red arrow).

The onset potential of the SnS photocathode shifted up to 0.4 V_RHE_, and the photocurrent density was also significantly increased to 28 mA cm^−2^ at 0 V_RHE_ (Figure 4b). As a result, the maximum ABPE increased from 1.70% to 3.01%, while the potential at which the maximum ABPE was observed also shifted from 0.115 to 0.186 V_RHE_, as shown in Figure S15, Supporting Information. The enhanced onset potential and fill factor could be attributed to the increased flat‐band potential in the presence of the Ga_2_O_3_ interlayer. To further support the enhanced photovoltage upon the Ga_2_O_3_ modification, we determined the open circuit photovoltage (OCP) of the photocathodes which is frequently used to reveal photovoltage enhancement. For each constructed electrode structure, the OCP is the subtracted value of open circuit voltage upon AM 1.5G illumination (OCV_light_) from open circuit voltage in dark (OCV_dark_, also known as the resting potential), representing the amount of the band bending at the time being with respect to that in the dark condition. As shown in Figure S16, Supporting Information, Pt/TiO_2_/Ga_2_O_3_/SnS/Au/FTO exhibited an OCP of 0.8 V, whereas Pt/TiO_2_/CdS/SnS/Au/FTO showed an OCP of 0.27 V. The degree of the band bending can be determined by the flat band potential in the photocathode/electrolyte junction as well as the minority carrier accumulation and the charge recombination. The enlarged band bending at the photocathode/electrolyte interface represents the enhanced electron−hole separation. In this regard, we clearly confirm that the conformal deposition of Ga_2_O_3_ on top of the TAA‐SnS surface effectively enlarges the band bending and thereby improves photovoltage. The increased photocurrent density can be attributed to the absence of photon blocking by the CdS overlayer. Figure S17a, Supporting Information shows the IPCE spectra of SnS photocathodes with either a CdS or Ga_2_O_3_ layer. The IPCE values at 300–550 nm decreased in the presence of the CdS layer. The reduced IPCE values indicate that the photons absorbed by CdS (E_g_ ≈2.4 eV, ≈520 nm) were unable to contribute to the photocurrent density. In contrast, no photons were lost in the case of the SnS photocathode with high band‐gap Ga_2_O_3_ layer (*E*_g_ ≈3.7 eV, ≈330 nm). In addition, Ga_2_O_3_‐applied SnS photocathode is capable of converting almost 100% of the absorbed light into the photocurrent, showing nearly theoretical photocurrent density (Figure S17b, Supporting Information).

We performed electrochemical impedance spectroscopy (EIS) to understand the charge transport mechanism in SnS photocathodes depending on different heterojunction. Figure 4c,d shows the Nyquist plots of the TAA‐SnS photocathodes containing either a CdS or Ga_2_O_3_ layer. The curves were fitted according to a Voight‐type equivalent circuit model (inset of Figure 4c), which consists of a series resistance (R_s_), three serial pairs of parallel combination of a resistance, and a constant‐phase element representing high (R_HF_, CPE_HF_), middle (R_MF_, CPE_MF_), and low (R_LF_, CPE_LF_) frequencies. It is speculated that the highest‐frequency RC element results from artifacts (e.g., interference by the reference electrode and ohmic resistances by electrolyte, wires, contacts, and others) without any physical relevance for the semiconductor junctions. The R_HF_ and CPE_HF_ were constant with respect to the applied potential regardless of the photocathode types.

Comparing the second arc at 0.3 V_RHE_ (Figure 4c,d), the large arc of CdS‐applied SnS photocathode was observed, while the Ga_2_O_3_‐applied SnS photocathode revealed a relatively small arc. The CdS‐interfaced SnS photocathode exhibited the high magnitude of the phase at 0.3 V_RHE_ in a low frequency region (<1 Hz), thereby indicating that no surface charge transfer occurs due to an onset potential lower than 0.3 V_RHE_, as shown in the Bode plot (Figure S18a, Supporting Information). However, in the same frequency region, the relatively small magnitude of the phase in the Ga_2_O_3_‐applied SnS photocathode was obtained, thus indicating the occurrence of surface charge transfer owing to a higher onset potential (≈0.4 V_RHE_) (Figure S18b, Supporting Information). In the middle frequency region, the maximum value of the phase associated with charge transport shifted to the frequency of ≈60 Hz by applying a negative potential to the CdS‐applied SnS photocathode. However, the maximum value of the phase shifted to a higher frequency (≈80 Hz) by applying a negative potential to the Ga_2_O_3_‐interfaced SnS photocathode. This indicates that the charge carriers are not recombined and the charge transport is accelerated as the band bends by the applied potential, since the charge transport time is inversely proportional to the frequency. The *R*_MF_ of the Ga_2_O_3_‐applied SnS photocathode (7.246 Ω·cm^2^) was lower than that of the CdS‐applied SnS photocathode (11.42 Ω·cm^2^) in 0 V_RHE_ (Table S1, Supporting Information). This implies that large band bending resulted from suitable band alignment with an *n*‐type Ga_2_O_3_ overlayer not only enables high photovoltage, but also improves the charge transport.

### 2.4 Tandem Device for Overall PEC Water Splitting

**Figure 5:**
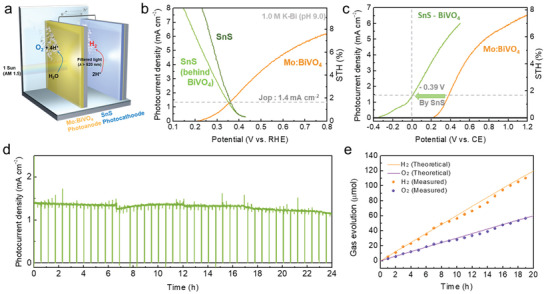
a) Schematic of a tandem device consisting of photoanode (NiFe/Mo:BiVO_4_/SnO_2_/FTO)—photocathode (Pt/TiO_2_/Ga_2_O_3_/SnS/Au/FTO) operating in pH 9.0 borate buffer electrolyte. b) *J*–*V* curve for the photocathode and photoanode; the operating point is marked for the tandem device (active area: 0.28 cm^2^). c) Two‐electrode measurements for photoanode–Pt counter electrode (C.E.) and photoanode–photocathode tandem devices (active area: 0.28 cm^2^). d) Operation stability under short circuit condition of the photoanode–photocathode tandem device. e) Time course curve of H_2_/O_2_ evolutions over the tandem device. The solid line indicates the calculated H_2_/O_2_ evolution, whereas the circular dots represent the H_2_/O_2_ evolution measured by gas chromatography. All analyses were performed in 1.0 m borate buffer at a scan rate of 10 mV s^−1^.

Since the band gap of SnS is 1.23 eV, which is appropriate for a D4 tandem device as a bottom electrode when combined with a top electrode of higher band gap,^[^ ^6^ ^]^ we fabricated tandem devices paired with BiVO_4_‐based photoanodes (*E*_g_ ≈ 2.4 eV), which have been widely used for high‐performance photocathode–photoanode tandem cells (**Figure** **5**a). The Mo:BiVO_4_ photoanode used here revealed good performance (≈6.4 mA cm^−2^ at 1.23 V_RHE_ and an onset potential of 0.2 V_RHE_ in a K‐Bi buffer solution with a pH of 9.0) as a top light absorber.^[^ ^38^, ^39^ ^]^ To achieve high efficiency, further optimization of the electrolyte was performed for the tandem cell containing two photoelectrodes involving opposite reactions. We tested various concentrations of borate buffer (0.1–1.0 m) to investigate its effect on both photoelectrodes (Figure S19, Supporting Information). The photocurrent of NiFe/Mo:BiVO_4_/SnO_2_/FTO (as a photoanode) showed almost no dependence on the electrolyte concentration. However, the Pt/TiO_2_/Ga_2_O_3_/SnS/Au/FTO photocathode exhibited better photocurrent density at a higher concentration (1.0 m K‐Bi buffer electrolyte) owing to high conductivity of the electrolyte. Since the precipitation of borate moieties occurred in an electrolyte concentration of more than 1.0 m, the tandem device was operated with a 1.0 m K‐Bi buffer solution as an optimal electrolyte.

As shown in Figure 5b, the operating point of the two photoelectrodes, as determined by the intersection of two *J*–*V* curves, was ≈1.4 mA cm^−2^ at 0.35 V_RHE_, which corresponded to an STH efficiency of 1.7%. Herein, we report the first demonstration of unbiased water splitting using an SnS photocathode in a tandem reactor. In addition, the polarization curve comparison of the Mo:BiVO_4_–Pt counter electrode and Mo:BiVO_4_–SnS demonstrated an anodic shift of ≈0.39 V induced by the SnS photocathode, thereby suggesting that the SnS photocathode provided a photovoltage of 0.39 V (Figure 5c). The BiVO_4_–SnS tandem cell had a photocurrent density of 1.4 mA cm^−2^ at 0 V against the counter electrode (i.e., unbiased condition), which is in good agreement with the estimated value obtained from the overlapped *J*–*V* curves shown in Figure 5b. This observation confirmed that the total photovoltage of 1.41 V (0.39 and 1.02 V from the SnS photocathode and the BiVO_4_ photoanode, respectively) is sufficient to implement unbiased overall water splitting. This results in both hydrogen and oxygen evolutions, while overcoming the thermodynamic potential requirement of 1.23 V. In comparison to previously reported PEC tandem devices, the total photovoltage of 1.41 V is a reasonable value considering the band gap. Therefore, an onset potential enhancement by Ga_2_O_3_ played a critical role in accomplishing the unbiased water splitting.

Photon absorption and conversion capability of the Mo:BiVO_4_–SnS tandem cell was verified through IPCE measurements. We compared Mo:BiVO_4_ and SnS in different applied potential conditions. The IPCE curves for both photoelectrodes at high applied bias condition near short circuit (i.e., 1.2 V_RHE_ for photoanode and 0 V_RHE_ for photocathode) are shown in Figure S20a, Supporting Information, while Figure S20b, Supporting Information shows the IPCE at actual operating bias (0.35 V_RHE_). In both cases, a wide‐range of photons could be efficiently utilized through synergetic absorption from Mo:BiVO_4_ (to 516 nm) and SnS (from 450 to 1020 nm). Both the photocathode and photoanode showed a high IPCE of ≈70% and ≈60%, respectively, under high bias condition. The photocurrent density estimated from the IPCE was 6.5 mA cm^−2^ for the Mo:BiVO_4_ photoanode, whereas those of the SnS photocathodes were 13 and 9.5 mA cm^−2^ behind or without Mo:BiVO_4_, respectively. These results are in good agreement with the photocurrents in the *J*–*V* curves of Figure S19, Supporting Information. In contrast, significantly lower IPCE values of Mo:BiVO_4_ and SnS were observed under the operating potential (0.35 V_RHE_), as shown in Figure S20b, Supporting Information. The photocurrent density calculated using the IPCE measurements of the Mo:BiVO_4_ and SnS photoelectrodes at 0.35 V_RHE_ was ≈1.4 mA cm^−2^, which is also analogous to the operating photocurrent, as determined in Figure 5b. The PEC tandem device consisting of the Mo:BiVO_4_ photoanode and SnS photocathode durably operated for over 24 h without any significant degradation in the photocurrent (Figure 5d). During the stability test of the tandem device, linear sweep voltammetry was performed every 6 h, and there was no noticeable decrease in the device performance (Figure S21, Supporting Information). Finally, the unbiased solar water‐splitting photocurrent was found to be stable, and the hydrogen/oxygen gases were evolved in a constant ratio of ≈6 µmol h^−1^ upon the illumination from the Mo:BiVO_4_–SnS tandem device (Figure 5e). The efficiency and stability of our SnS‐based tandem devices are highly comparable to the best‐performing tandem devices based on other photocathodes, as shown in Figure S22, Supporting Information. Our results demonstrated the first successful approach for exploiting a low‐cost SnS‐based photoelectrode solar water‐splitting device.

## 3 Conclusions

We have demonstrated a high‐performance photocathode using a low‐cost SnS semiconductor, which has a small Eg (≈1.2 eV), good optoelectronic properties, and no secondary phase. The precursor polarity‐controlled ink enabled to obtain a compact and well‐oriented SnS nanoplates with extended (101) facets. The resulting SnS based photocathode revealed the highest photocurrent density of up to 23 mA cm^−2^ with the onset potential of 0.3 V_RHE_ by avoiding recombination and accelerating charge separation through the favorable (101) facet oriented SnS nanoplates. Instead of CdS, inserting a Ga_2_O_3_ layer between SnS and TiO_2_ further improved the onset potential to 0.4 V_RHE_ and consequently enhanced the ABPE up to 3.01%. By combining the photocathode with a Mo:BiVO_4_ photoanode, unbiased solar water splitting was successfully achieved with remarkable efficiency (≈1.7%) and long‐term stability of 24 h. This PEC performance significantly exceeds the previously reported SnS photocathodes, which is comparable to other expensive multicomponent chalcogenide photocathodes. Considering the relative short development history, it is expected that rapid advance of SnS photocathodes will lead to higher efficiency in the foreseeable future and our results clearly demonstrate the feasibility of standalone solar water splitting through low cost photocathode‐photoanode tandem system. We believe that the emerging SnS photoelectrode can establish a new benchmark for practical solar fuel production.

## 4 Experimental Section

### Preparation of SnS Inks

The SnS seed ink was prepared by weighing 3 mmol of SnCl_2_·2H_2_O (Sigma Aldrich, 98%, USA) and 3 mmol of sulfur powder (Sigma Aldrich, 99.98%, USA) in a N_2_‐filled glovebox. 2 mL of 2‐mercaptoethanol (Sigma Aldrich, 99%, USA) and 8 mL of ethylenediamine (Sigma Aldrich, 99.5%, USA) were added to dissolve the precursor mixture. Then, 10 mL of 2‐methoxyethanol (2ME, Sigma Aldrich, ≥99.0%, USA) was added, followed by stirring and aging at 60 °C for overnight. Three different SnS absorber inks were prepared by using different sulfur precursor and the identical amount of SnCl_2_·2H_2_O (15 mmol). 0.76 g of thiourea (10 mmol, Sigma Aldrich, 98%, USA), 0.75 g of thioacetamide (10 mmol, Sigma Aldrich, 99.0%, USA), and 1.21 g of L‐cysteine (10 mmol, Sigma Aldrich, 97%, USA) were separately dissolved in 10 mL of 2ME as well as the addition of SnCl_2_·2H_2_O, followed by stirring at 60 °C for 30 min in a N_2_‐filled glovebox.

### Fabrication of SnS Absorber

As previously reported,^[^ ^21^ ^]^ the SnS absorber ink was coated after SnS seed layer formation from the seed ink. First, the 70 nm thick Au‐coated FTO glass substrates (TEC‐8, Pilkington, UK) were ultrasonically cleaned in ethanol for 15 min, followed by UV treatment for 15 min. The SnS seed ink was spin‐coated onto the substrates at 500 rpm for 5 s and then 3500 rpm for 25 s. After the spin‐coating method, the samples were instantly annealed on a hotplate at 180 and 300 °C for 3 min in a N_2_‐filled glovebox. After sufficient cooling, the SnS absorber inks were spin‐coated on top of the SnS seed layer/Au/FTO. The spin‐coating process was conducted four times, followed by annealing in the same way and then post‐annealing at 350 °C for 20 min in a N_2_ atmosphere.

### Characterization of SnS Nanostructures

The surface morphologies and cross‐sectional images of the TU‐ and TAA‐SnS were analyzed using a field‐emission SEM (JSM‐7001F, JEOL, Japan). Phase analysis of the SnS nanostructures was performed via XRD measurements (MiniFlex 600, Rigaku, Tokyo, Japan) with Cu K*α* radiation (*λ* = 0.15406 nm) to investigate the crystallinity of the SnS. Raman scattering analysis of the TU‐ and TAA‐SnS was performed using a LabRam Aramis spectrometer (Horiba, Japan) equipped with a charge‐coupled device camera at room temperature. The excitation source was the 532‐nm line of an Ar‐ion laser with a beam intensity of 1.0 mW. The liquid Raman spectra (LabRam Aramis, Horiba, Japan) for the step‐wise sequentially prepared SnS absorber inks were obtained using an Ar‐ion laser beam at an exciting radiation wavelength of 514.5 nm. The absorbance spectra were observed at room temperature using a UV−vis spectrophotometer (V‐670, JASCO, Easton, MD, USA) equipped with an integrating sphere. The Tauc plot was obtained to determine the optical band gaps of SnS using the absorbance spectra. The c‐AFM (SPA 400, Seiko Instruments, Inc., Chiba, Japan) in an area of 2 µm × 2 µm under an applied bias of 2.0 V was performed using a Rh‐coated cantilever to investigate the electrical properties of the SnS films. The energy level of each semiconductor was analyzed by ultraviolet photoelectron spectroscopy (UPS, Axis‐NOVA, and Ultra DLD, UK) under He I radiation (21.21 eV). The secondary‐electron cutoff (*E*_cutoff_) obtained via extrapolation to the linear part of the binding‐energy edge. The Fermi level (*E*_F_) of each material was calculated using the following equation:

(3) $$E_{F}=E_{\mathrm{cutoff}}-21.21\;\mathrm{eV}\;\left(\mathrm{under}\;\mathrm{He}\;I\;\mathrm{radiation}\right)$$

The valence‐band edge (*E*_edge_) represents the difference between *E*_VBM_ and *E*_F_ for each material. Using the *E*_edge_ values, the definite VBM level of the samples was calculated as follows:

(4) $$E_{\mathrm{VBM}}=E_{F}+E_{\mathrm{edge}}$$

### Fabrication of SnS Photocathodes

For the Pt/TiO_2_/CdS/SnS/Au/FTO device, the *n*‐type CdS layer was deposited via chemical bath deposition. The SnS samples were treated in a bath solution of CdSO_4_ (Sigma Aldrich, 99.99%, USA), thiourea, deionized water, and NH_4_OH (Duksan, 28 wt%, Korea) at 60 °C for 3 min with stirring. After the CdS deposition, the samples were rinsed with deionized water. Additionally, an *n*‐type TiO_2_ layer was deposited via ALD of tetrakis(dimethylamido) titanium (IV) (TDMAT, Easychem, Korea) and H_2_O as Ti and O sources, respectively. The deposition temperature was 120 °C, and the TDMAT was evaporated at 75 °C. For the Pt/TiO_2_/CdS/SnS/Au/FTO photocathodes, TiO_2_ was deposited via 300 cycles, yielding a thickness of ≈17 nm. Each ALD cycle consisted of 3 s of TDMAT pulses followed by 10 s of high‐purity N_2_ purging and 2 s of H_2_O pulses followed by 10 s of N_2_ purging. Finally, a Pt co‐catalyst was deposited on the TiO_2_ by using a 108 Auto Sputter Coater (Ted Pella, Redding, CA, USA) for 120 s under the current of 10 mA and 0.1 mbar of Ar pressure. For the Pt/TiO_2_/Ga_2_O_3_/SnS/Au/FTO, the Ga_2_O_3_ layer was deposited by ALD of bis (µ‐dimethylamino) tetrakis (dimethylamino) digallium (Strem Chemicals, US) and H_2_O as Ga and O sources, respectively. The deposition temperature was 160 °C, and the TDMAT was evaporated at 117 °C. The Ga_2_O_3_ layer was deposited via 100 cycles yielding a thickness of ≈10 nm. The TiO_2_ and Pt were deposited in the same conditions as mentioned above.

### Fabrication of Mo:BiVO_4_ Photoanodes

Cleaned FTO glass substrates with the size of 1.5 cm × 1 cm and carbon crucibles with SnO_2_ sources (Taewon Scientific Co., 99.99%) were placed in an e‐beam evaporator. Evacuating the chamber to a pressure of 2 × 10^−6^ torr, SnO_2_ adhesion layer with ≈50 nm thickness was deposited on FTO glass substrates. 3‐µm‐thick SnO_2_ nanorods (NRs) were formed by the glancing angle deposition (glancing angle of 85°, rotation speed of 80 rpm), followed by annealing at 550 °C for 2 h. BiOI was electrodeposited on the SnO_2_ NRs in three‐electrode cell with Ag/AgCl (3 m NaCl) reference electrode and Pt counter electrode. The plating solution was based on 20 mL of ethanol (Daejung, 99.9%) containing 46 mm *p*‐benzoquinone (JUNSEI, 98%) and 50 mL of an aqueous solution containing 400 mm KI (Daejung, 99.5%), 15 mm Bi(NO_3_)_3_∙5H_2_O (Junsei, 98%), and 30 mm lactic acid (Aldrich, 85%). After mixing the plating solution for 30 min, nucleation of BiOI was conducted at −0.35 V versus Ag/AgCl for 20 s, followed by the growth of BiOI at −0.10 V versus Ag/AgCl for 360 s. For the conversion into Mo‐doped BiVO_4_ (Mo:BiVO_4_), 10 µL of an aqueous solution containing 100 mm Na_2_MoO_4_ (Daejung, 98.5%) and 5 mL of DMSO (Kanto, 98%) containing 200 mm VO(acac)_2_ (Aldrich, 98%) were mixed. BiOI/SnO_2_ NRs soaked by 50 µL of the mixing solution were annealed in air at 450 °C for 2 h, followed by removing the residual V_2_O_5_ in a 1 m NaOH solution (Daejung, 99%) for 10 min. NiFe alloy pellets (50 wt%: 50 wt%) were placed in carbon crucibles and subjected to e‐beam evaporation. NiFe oxygen evolution catalyst with ≈5–10 nm thickness was deposited on Mo:BiVO_4_/SnO_2_ NRs with a deposition rate of 0.2 Å s^−1^.

### PEC Measurements

PEC measurements were conducted using a potentiostat (SI 1287, Solartron, UK) in a 0.5 m aqueous H_2_SO_4_ solution (pH 1) with a three‐electrode configuration (Pt wire as the counter electrode and an Ag/AgCl/KCl (4 m) as reference electrode). PEC measurements for tandem devices were carried out in a 1.0 m potassium borate (K‐Bi) buffer (pH 9.5) without the hole scavenger. A commercial AM 1.5G solar simulator and a Si reference cell (Newport Corporation, USA) were utilized for the simulated sunlight and 1‐sun calibration, respectively. For all PEC measurements, the applied potentials were based on the RHE scale for comparison with other reports. The following equation was used to convert the potential:

(5) $$E_{\mathrm{RHE}}=E_{\mathrm{Ag}/\mathrm{AgCl}}+0.059\;\mathrm{pH}+0.197$$

EIS was conducted using the same potentiostat, along with a frequency analyzer (1260, Solartron, Leicester, UK). The series and polarization resistances of the SnS photocathodes were measured in the frequency range of 300 kHz to 0.1 Hz under 1‐sun illumination at 0 V versus RHE with an alternating‐current amplitude of 10 mV. IPCE and IMVS measurements were conducted using an electrochemical workstation (Zennium, Zahner, Germany) and a potentiostat (PP211, Zahner, Germany) with a monochromatic light source (TLS03, Zahner). IMVS was conducted in an open‐circuit condition with a 10% perturbation of the light intensity. The frequency of the light modulation was swept from 100 kHz to 0.1 Hz. Gas chromatography (6500GC system, YL Instrument, Anyang, Korea) was performed using a pulsed discharge detector and a molecular sieve column to analyze the H_2_ evolution. All the connections of the device were completely sealed with rubber bulkheads to prevent gas leakage from the quartz reactor.

## Conflict of Interest

The authors declare no conflict of interest.

## Supporting information

Supporting Information

## Data Availability

Research data are not shared.
