# Supplementary material for: Crystal Facet‐Controlled Efficient SnS Photocathodes for High Performance Bias‐Free Solar Water Splitting
Source: Adv Sci (Weinh). 2021 Sep 8;8(21):2102458. doi: 10.1002/advs.202102458 (PMC8564457; doi:10.1002/advs.202102458)
Supplement: Supplementary file 1 — Supporting Information [file ADVS-8-2102458-s001.pdf]

© 2021 Wiley-VCH GmbH

ADVANCED SCIENCE
Open Access

Supporting Information

for *Adv. Sci.*, DOI: 10.1002/advs.202102458

Crystal Facet-Controlled Efficient SnS Photocathodes for High Performance Bias-Free Solar Water Splitting

Hyungsoo Lee,[†] *Jin Wook Yang,*[‡] *Jeiwan Tan,*[†] *Jaemin Park,*[†] *Sang Gi Shim,*[†] *Young Sun Park,*[†] *Juwon Yun,*[†] *Kyungmin Kim,*[†] *Ho Won Jang*,*[‡] *and Jooho Moon**[,†]

Supporting Information

Crystal Facet-Controlled Efficient SnS Photocathodes for High Performance Bias-Free Solar Water Splitting

Hyungsoo Lee,[†] *Jin Wook Yang,*[‡] *Jeiwan Tan,*[†] *Jaemin Park,*[†] *Sang Gi Shim,*[†] *Young Sun Park,*[†] *Juwon Yun,*[†] *Kyungmin Kim,*[†] *Ho Won Jang**,[‡] *and Jooho Moon**,[†]

*E–mail: hwjang@snu.ac.kr, jmoon@yonsei.ac.kr;

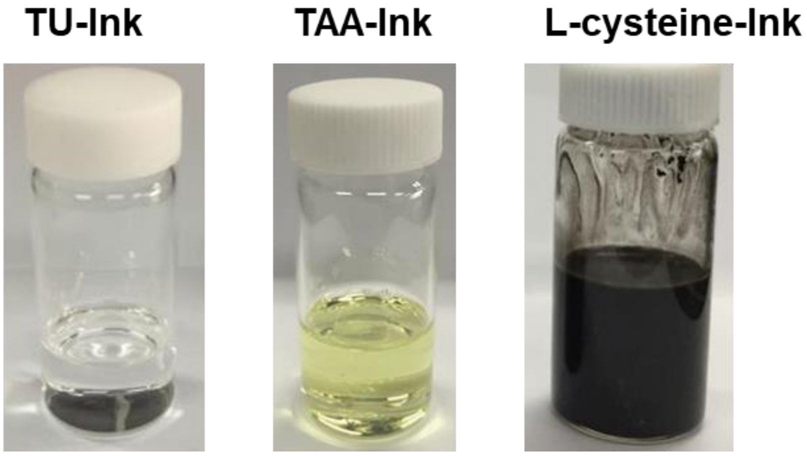

Figure S1. Photographs of precursor inks using TU, TAA and L-cysteine as different sulfur precursor.

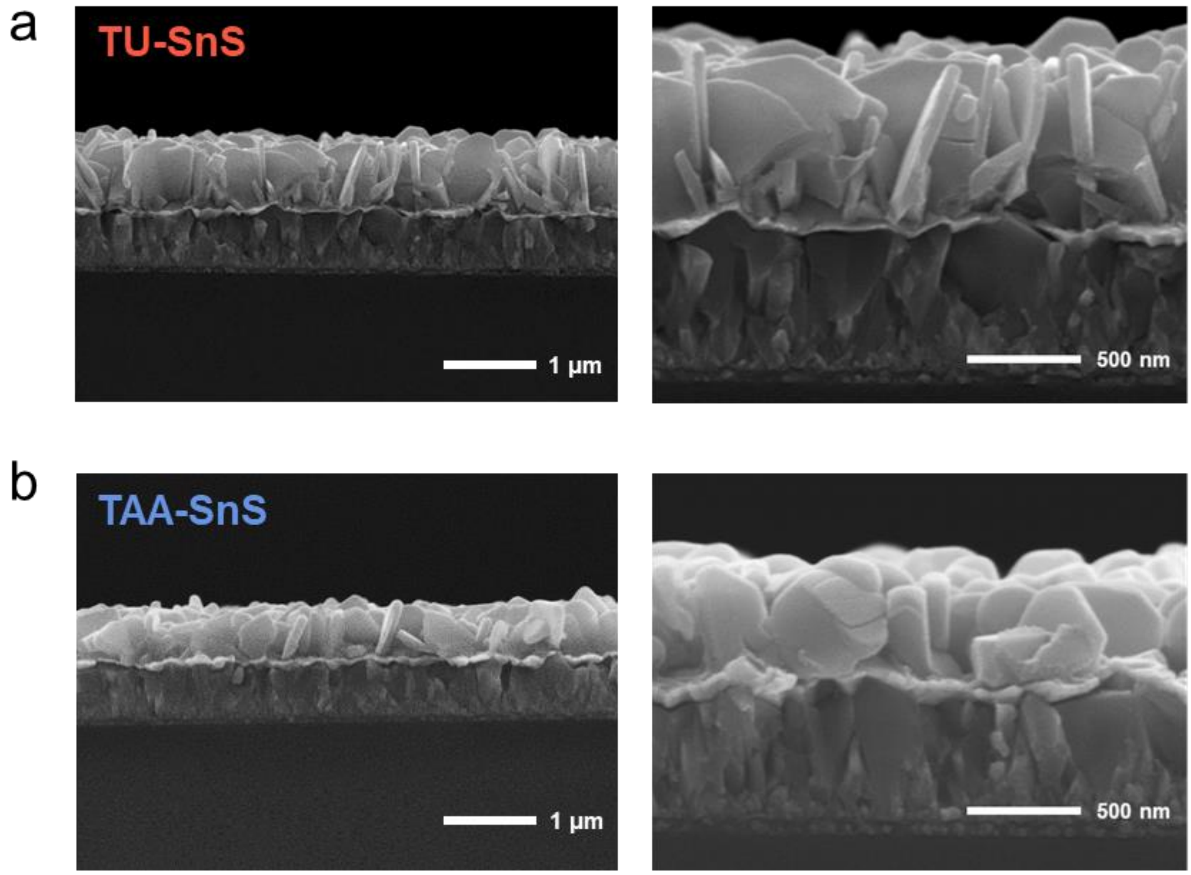

Figure S2. Cross-sectional SEM images of b) TU-SnS and d) TAA-SnS layers.

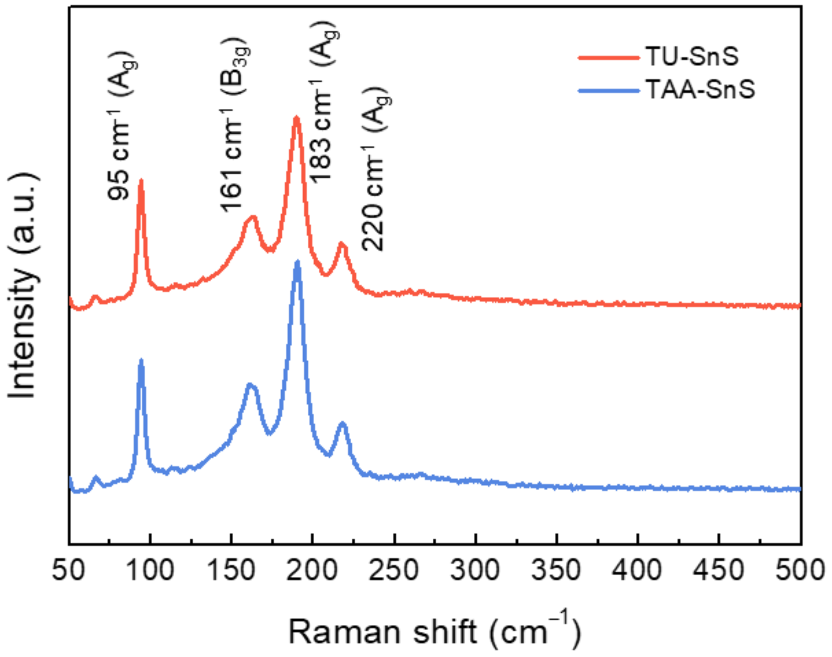

Figure S3. Raman spectra for the TU-SnS (red) and TAA-SnS (blue).

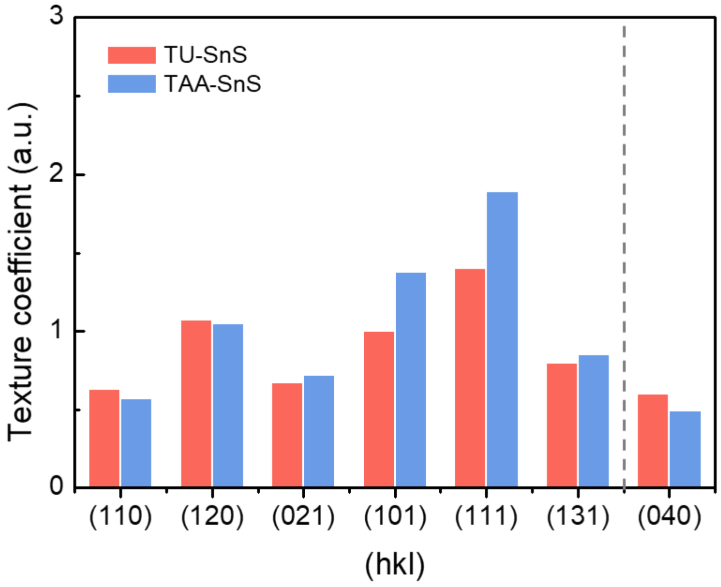

Figure S4. Texture coefficients of selected diffraction peaks for different SnS nanostructured layers derived by either TU-ink or TAA-ink.

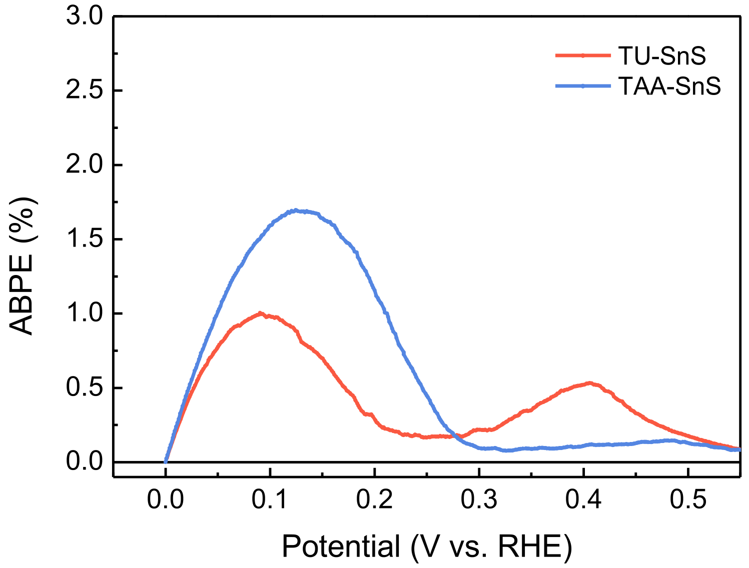

Figure S5. ABPE curves of Pt/TiO_2/CdS/SnS/Au/FTO photocathode based on either TU-SnS or TAA-SnS under solar simulated AM 1.5G irradiation in 0.5 M H_2SO_4 electrolyte (pH ≈ 1).

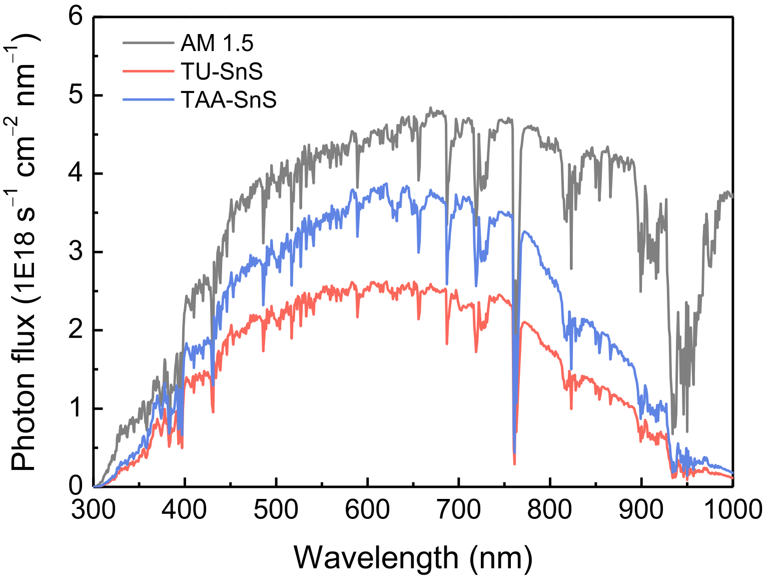

Figure S6. The photon flux of AM 1.5G spectrum and calculated photon flux curves for each photoelectrode against AM 1.5G spectrum.

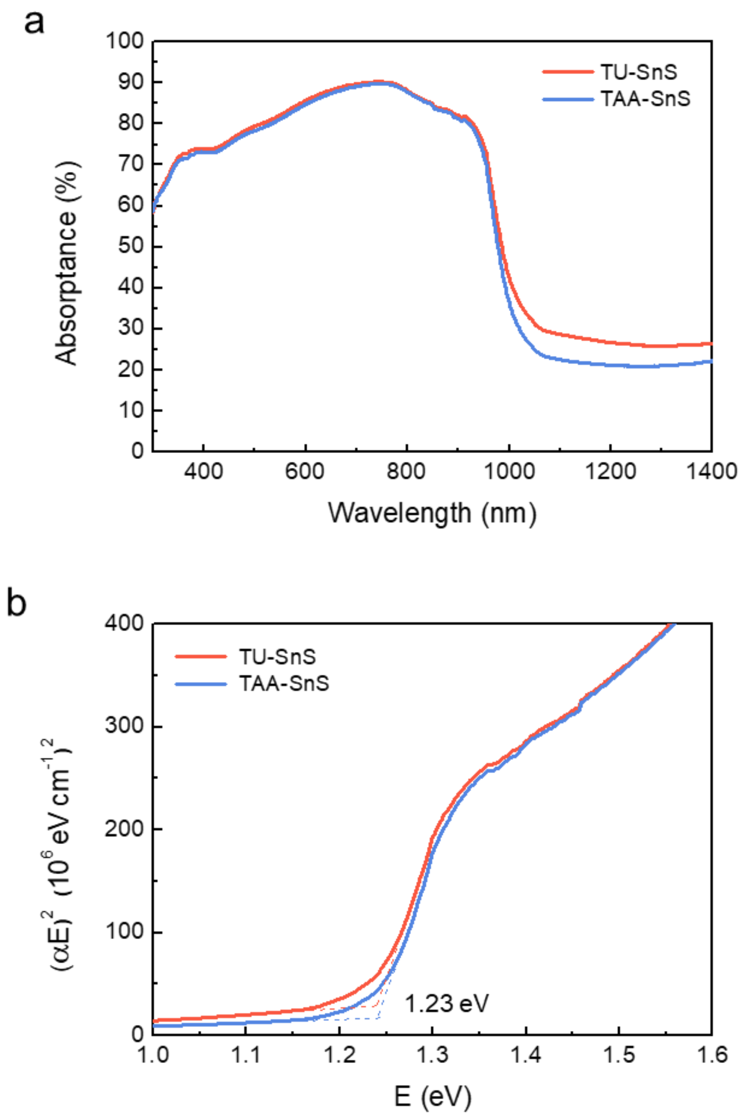

Figure S7. a) UV–vis absorbance and b) Tauc plots for TU- and TAA-SnS to determine the optical band gaps.

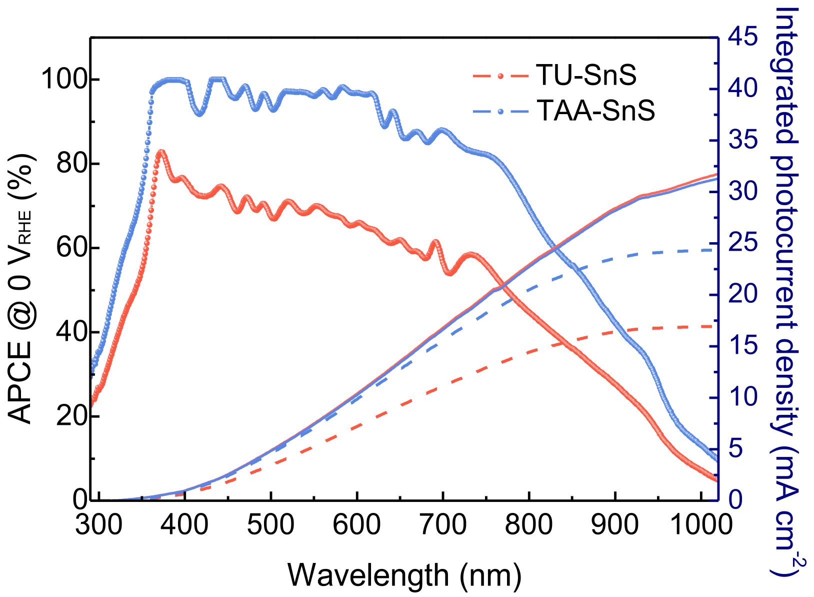

Figure S8. Absorbed photon-to-current efficiency (APCE) for both photocathodes in a 0.5 M aqueous H_2SO_4 solution biased at 0 V_{RHE}. Integration of the APCE values with the solar AM 1.5 spectrum gives a photocurrent density (dotted line). The calculated theoretical maximum photocurrent density is also shown as a solid line when assuming 100% APCE.

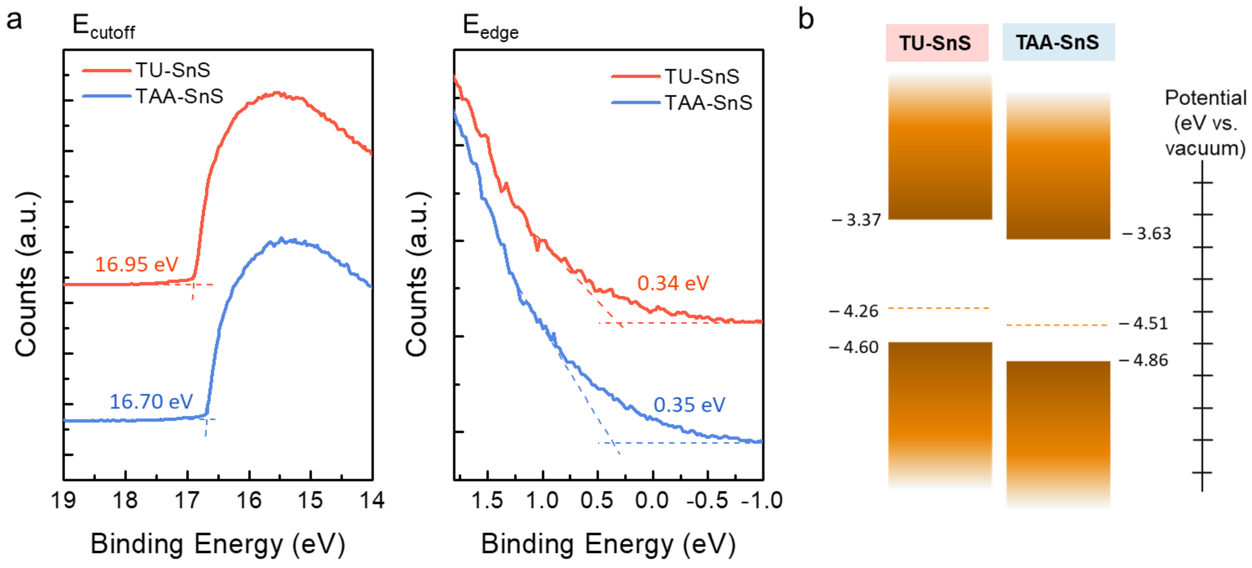

Figure S9. a) UPS for the TU- and TAA-SnS obtained using He I radiation at 21.21 eV to determine E_{cutoff} and E_{edge}. b) Schematic band diagram showing the relative energy positions of CBM, VBM, and E_F for the two different SnS before equilibrium.

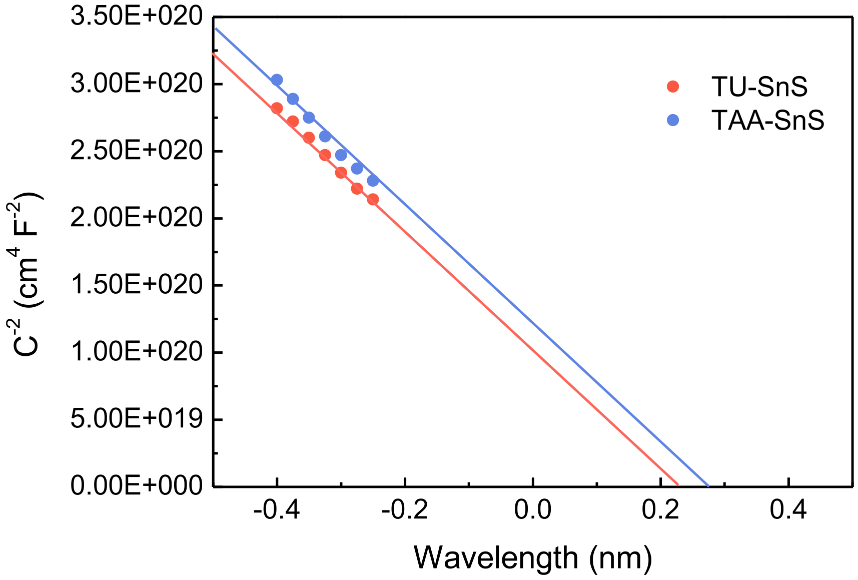

Figure S10. Mott–Schottky plots for TU-SnS and TAA-SnS in a 0.5 M aqueous H_2SO_4 solution at pH 1.

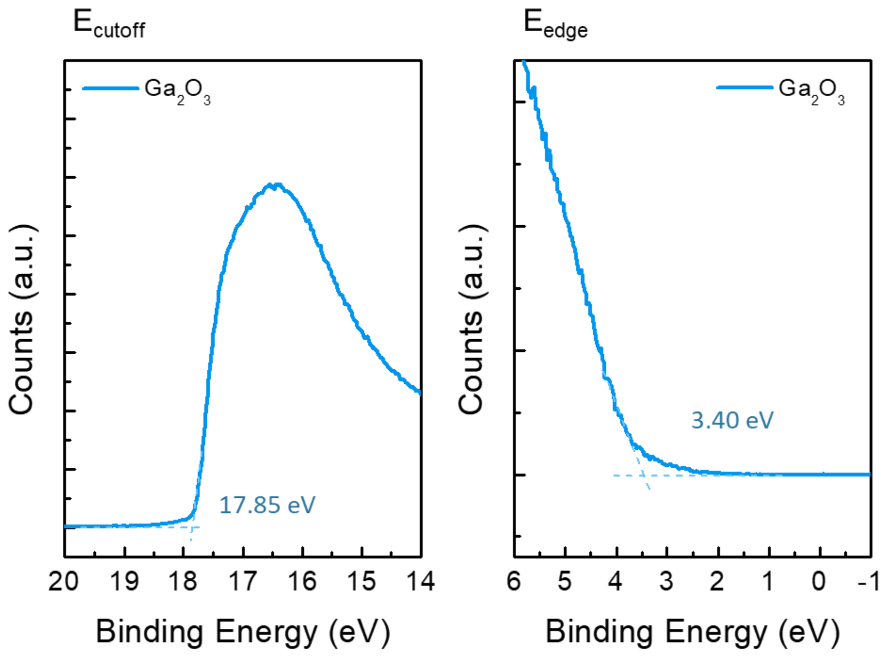

Figure S11. UPS for the Ga_2O_3 obtained using He I radiation at 21.21 eV to evaluate E_{cutoff} and E_{edge}.

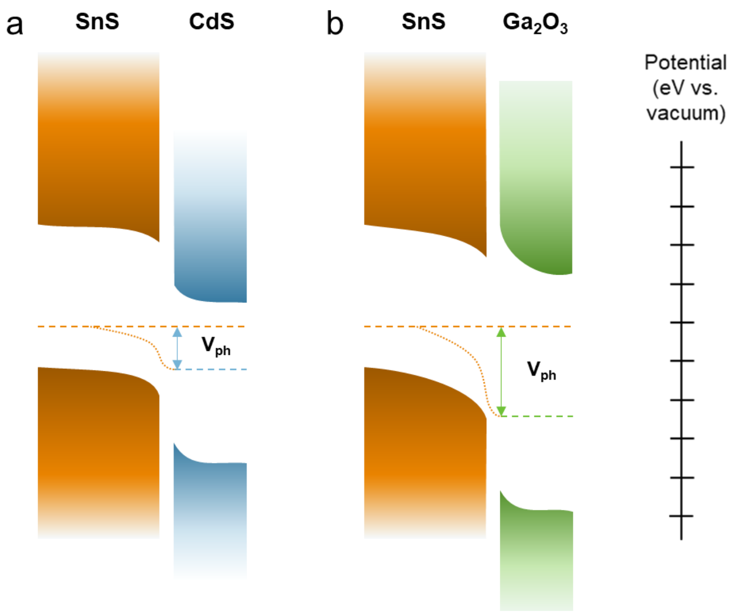

Figure S12. Illuminated equilibrium states in a) CdS/SnS and b) Ga_2O_3/SnS heterojunctions, showing quasi-Fermi level splitting (V_{ph})

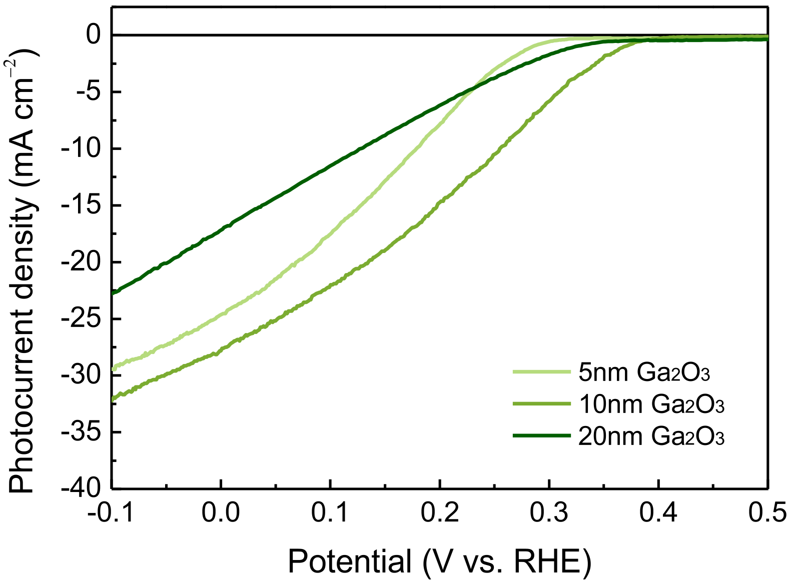

Figure S13. J-V curves of Pt/TiO_2/Ga_2O_3/SnS/Au/FTO photocathodes as a function of Ga_2O_3 overlayer thickness under solar simulated AM 1.5G irradiation in 0.5 M H_2SO_4 electrolyte (pH ≈ 1)

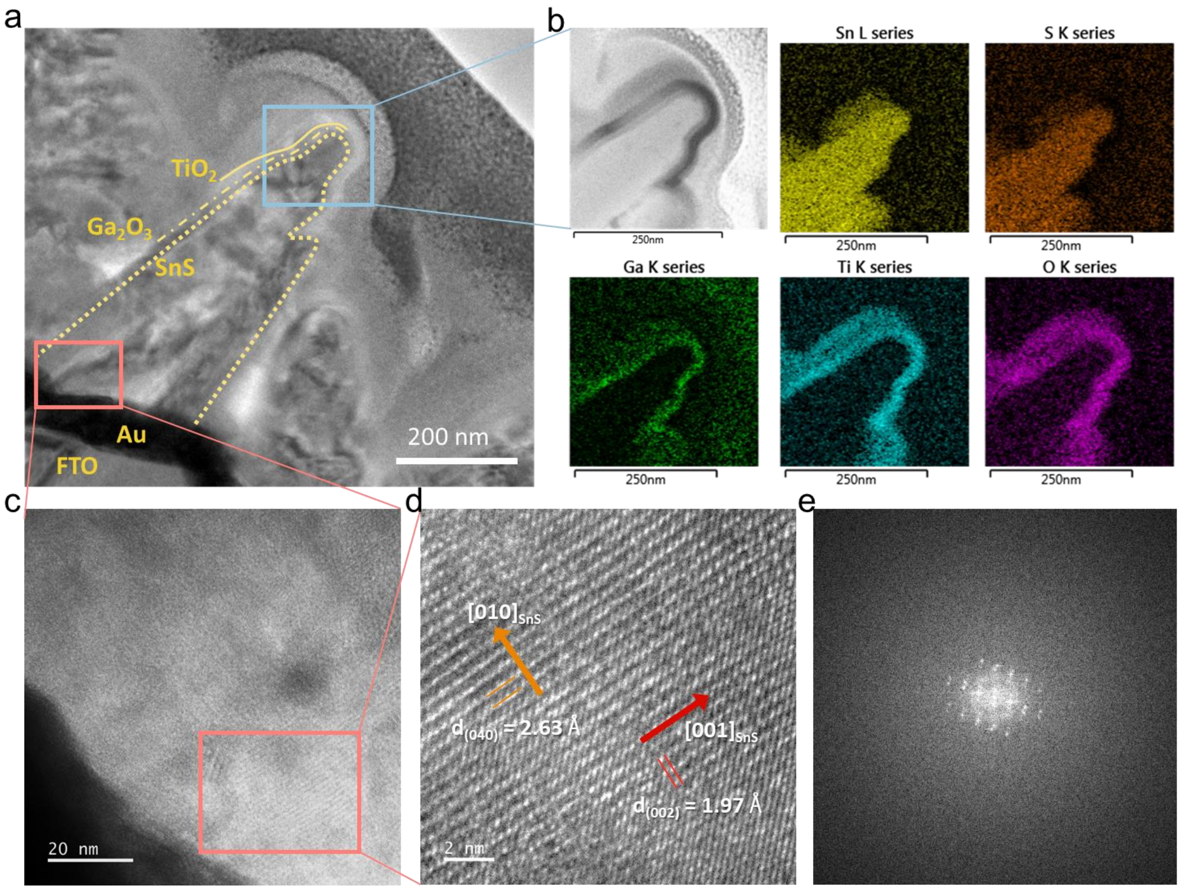

Figure S14. a) Cross-sectional TEM images of the Pt/TiO_2/Ga_2O_3/SnS/Au/FTO photocathode. b) Selected TEM image of the Pt/TiO_2/Ga_2O_3/SnS/Au/FTO photocathode (blue box) and the corresponding STEM-EDS elemental mapping images for Sn, S, Ga, Ti and O, respectively. c) HRTEM image for the selected region of Pt/TiO_2/Ga_2O_3/SnS/Au/FTO photocathode (red box) as well as d) the corresponding lattice fringes and e) diffraction patterns via fast Fourier transform of SnS.

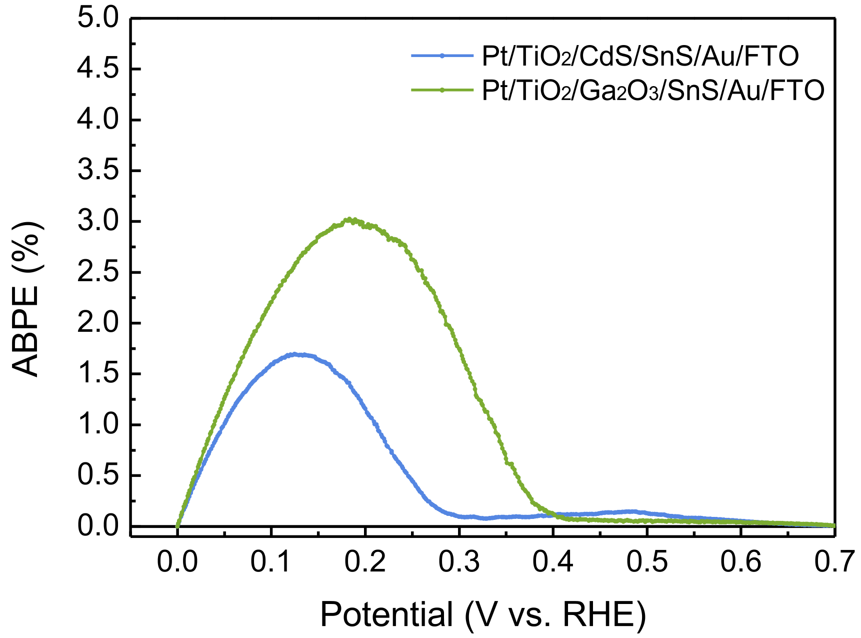

Figure S15. ABPE curves of Pt/TiO_2/CdS/SnS/Au/FTO and Pt/TiO_2/Ga_2O_3/SnS/Au/FTO photocathodes under solar simulated AM 1.5G irradiation in 0.5 M H_2SO_4 electrolyte (pH ≈ 1).

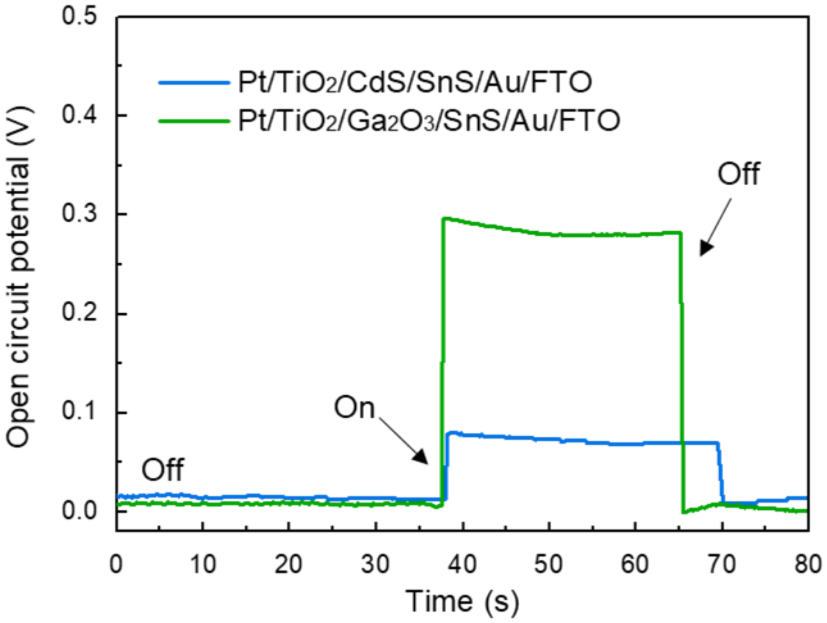

Figure S16. OCP values of Pt/TiO_2/CdS/SnS/Au/FTO and Pt/TiO_2/Ga_2O_3/SnS/Au/FTO photocathodes in a 0.5 M aqueous H_2SO_4 solution at pH 1 under AM 1.5G illumination and in dark.

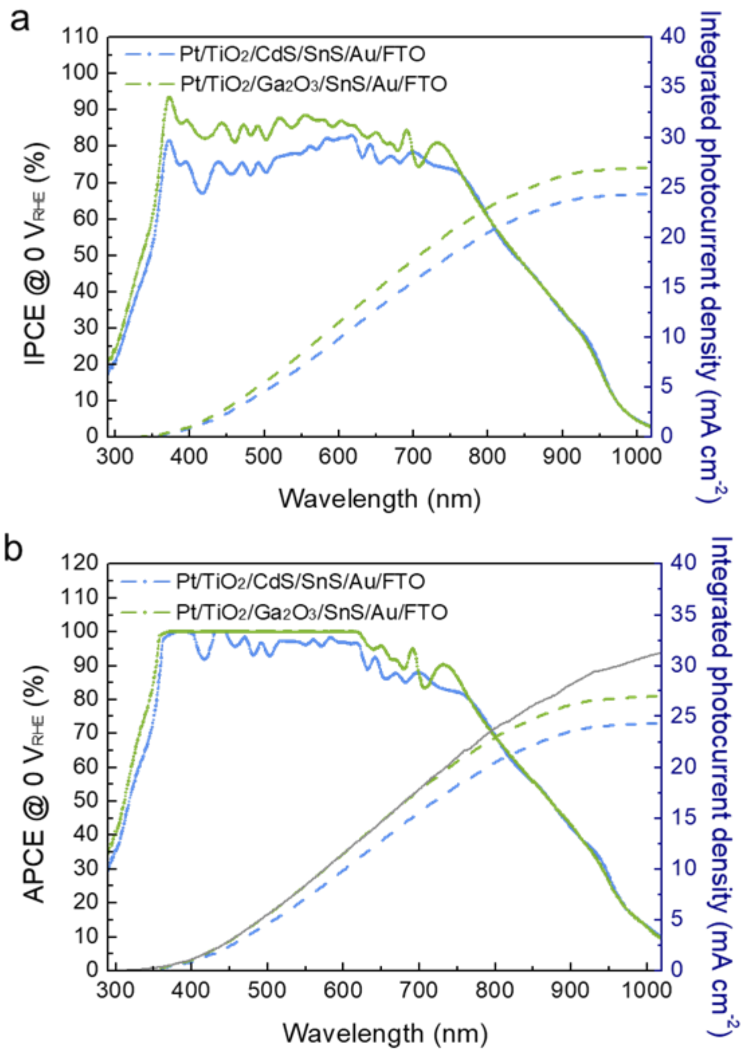

Figure S17. a) IPCE and b) APCE spectra of Pt/TiO_2/CdS/SnS/Au/FTO and Pt/TiO_2/Ga_2O_3/SnS/Au/FTO photocathodes at 0 V_{RHE} under 1-sun illumination in H_2SO_4 electrolyte (pH ≈ 1).

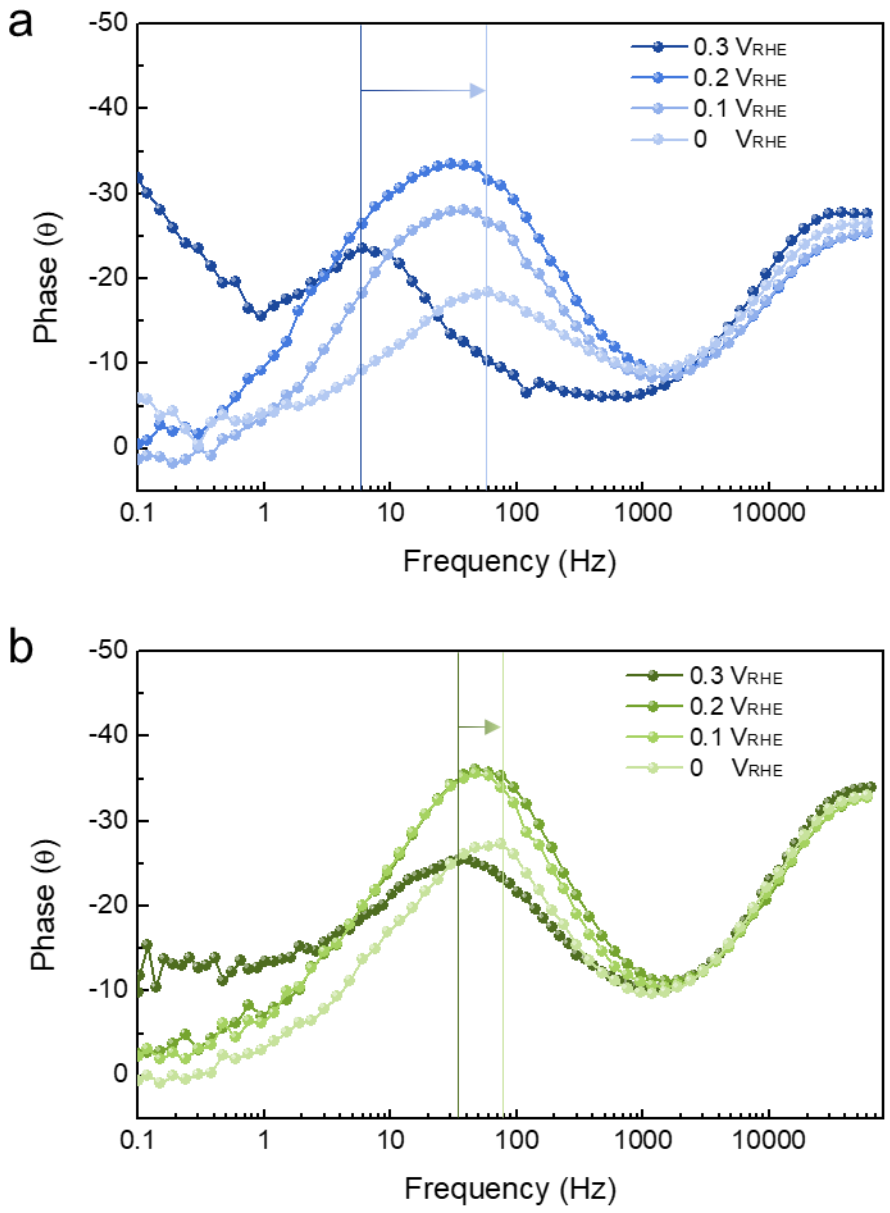

Figure S18. Bode plots of the EIS spectra for a) Pt/TiO_2/CdS/SnS/Au/FTO and b) Pt/TiO_2/Ga_2O_3/SnS/Au/FTO photocathodes at different applied bias ranging from 0.3 to 0 V_{RHE}.

Table S1. Area-specific resistance values and CPEs obtained by deconvoluting the EIS spectra at 0 V_{RHE}.

Photocathode	R_S ($\Omega\cdot cm^2$)	R_{HF} ($\Omega\cdot cm^2$)	CPE_{HF} (F s^{n-1} cm^{-2})	R_{MF} ($\Omega\cdot cm^2$)	CPE_{MF} (F s^{n-1} cm^{-2})
$Pt/TiO_2/CdS/SnS/Au/FTO$	1.58	4.625	2.032×10^{-5} (n = 0.78)	11.42	1.383×10^{-3} (n = 0.79)
$Pt/TiO_2/Ga_2O_3/SnS/Au/FTO$	1.44	4.296	1.929×10^{-5} (n = 0.81)	7.246	1.335×10^{-3} (n = 0.82)

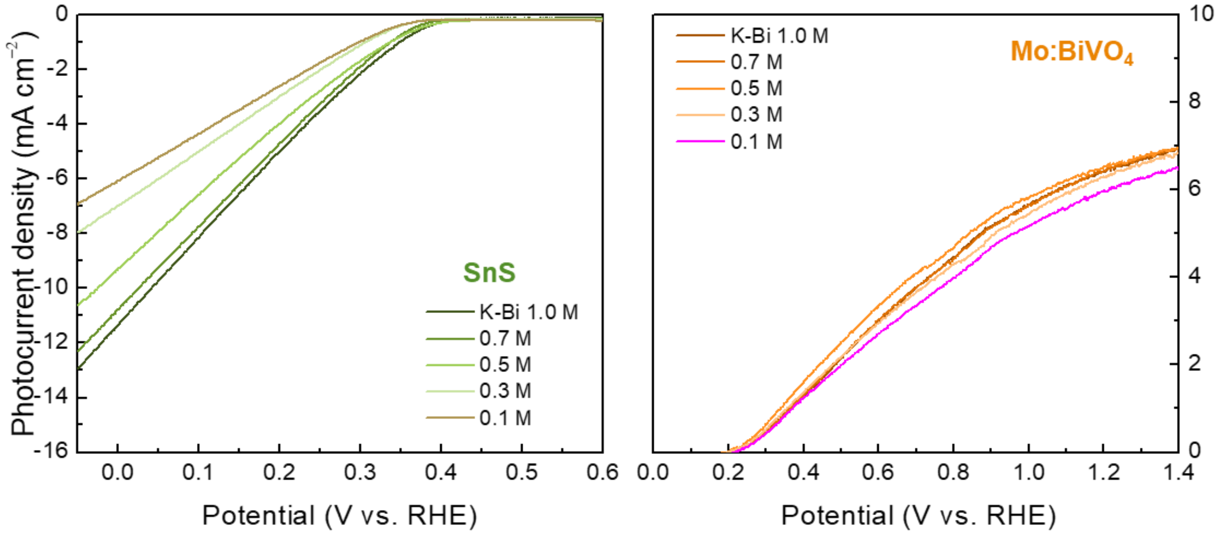

Figure S19. J–V measurement to determine the optimum electrolyte concentration for the SnS photocathodes and Mo:$BiVO_4$ photoanodes under borate buffer electrolyte (pH ≈9.0).

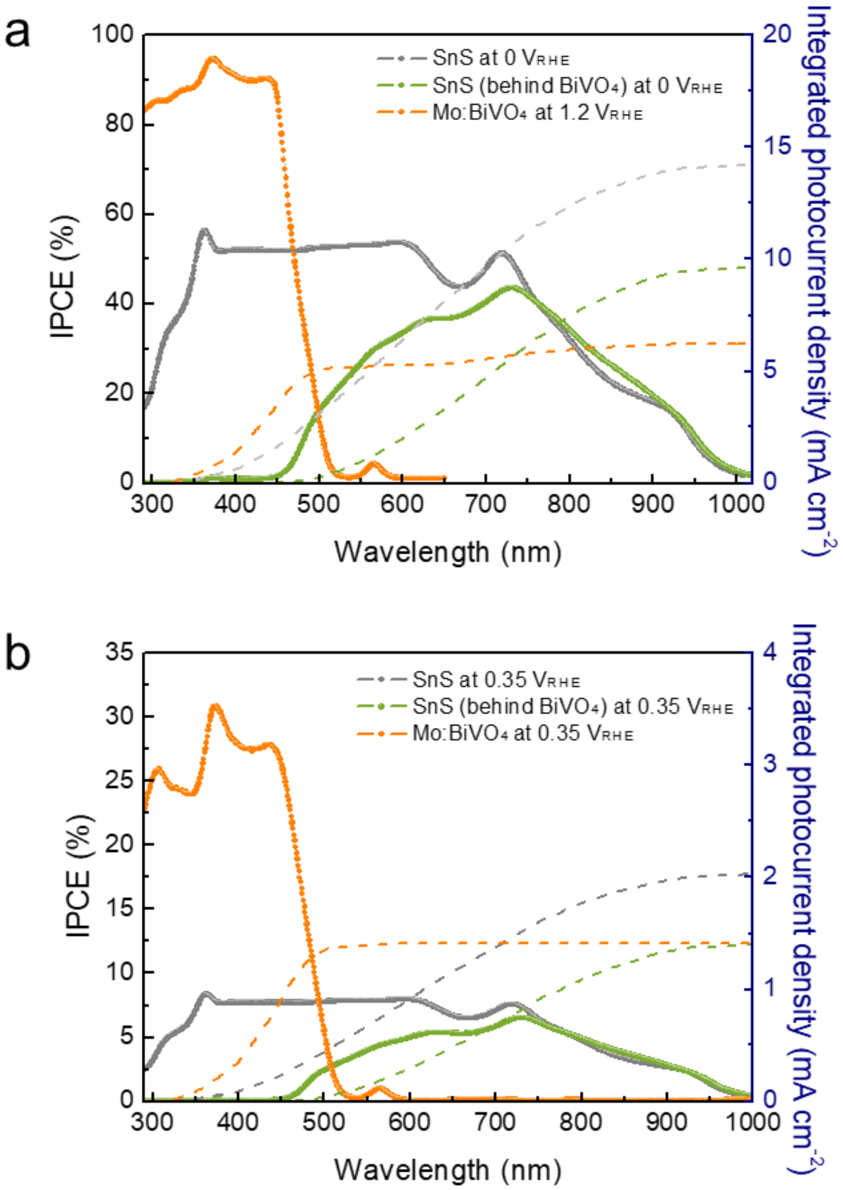

Figure S20. IPCE and calculated photocurrent density a) at 0 V_{RHE} for SnS photocathode and at 1.2 V_{RHE} for Mo:$BiVO_4$ photoanode and b) at 0.35 V_{RHE} (operating potential) for both photoelectrodes.

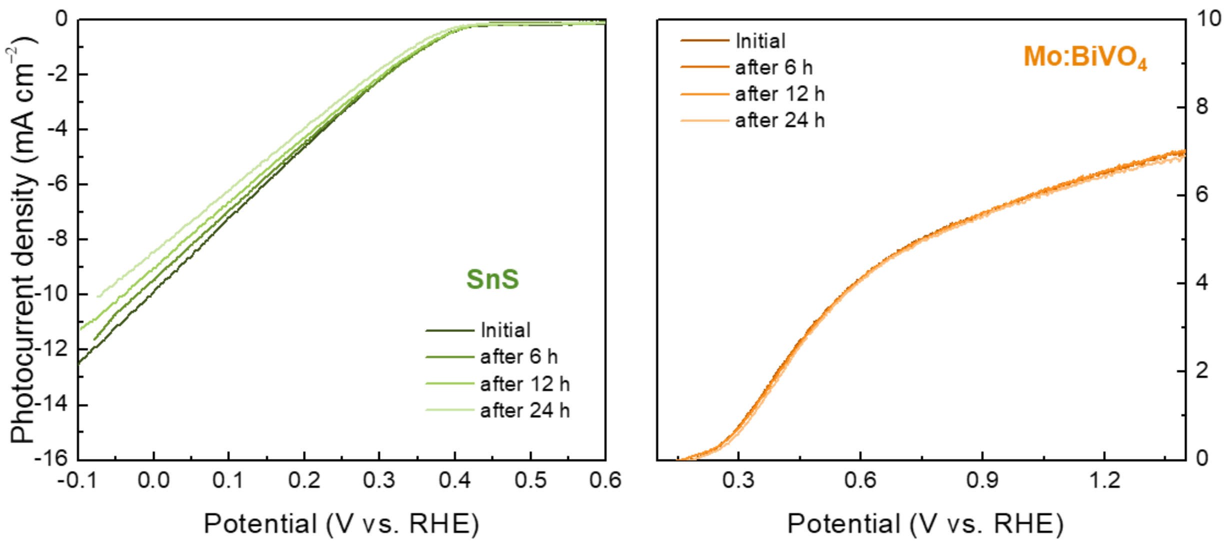

Figure S21. J–V curves for the SnS photocathodes and Mo:$BiVO_4$ photoanodes as a function of operation times, *i.e.*, after 6, 12, 24 h of duration for PEC tandem device under 1.0 M borate buffer (pH ≈9.0).

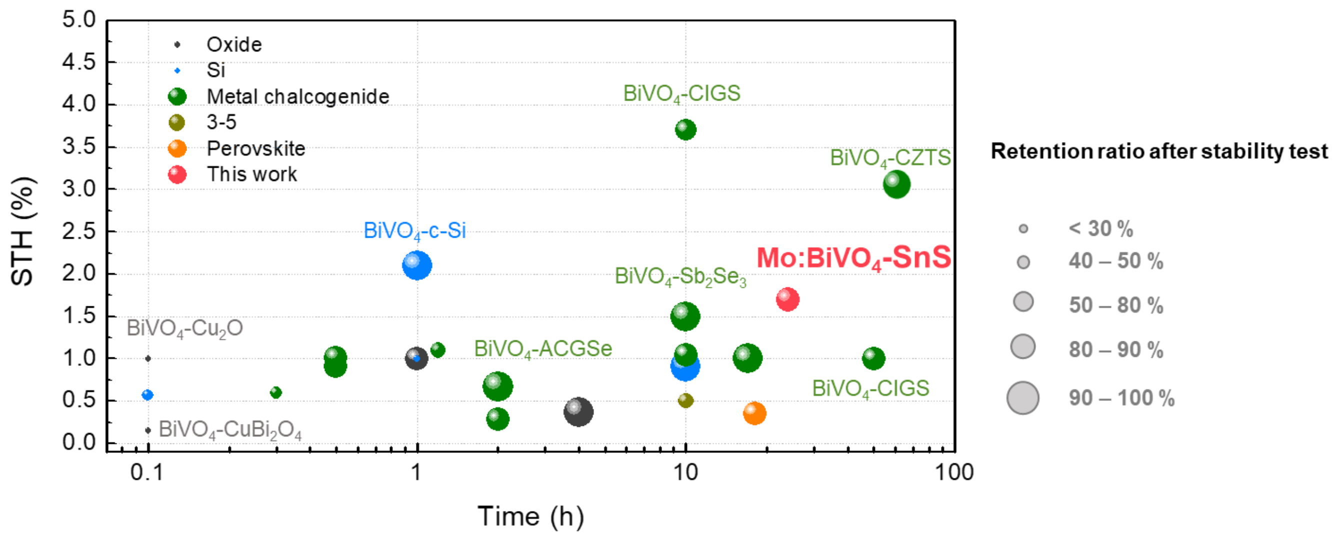

Figure S22. Efficiency and stability comparison chart for photoanode–photocathode D4 tandem devices whose STH and their operation duration were reported in recent years. The gray-colored circles on the right side represent the photocurrent retention ratio after stability test.
